# Effect of the Chronic Kidney Disease—Peritoneal Dialysis (CKD-PD) App on Improvement of Overhydration Treatment in Patients on Peritoneal Dialysis: Randomized Controlled Trial

**DOI:** 10.2196/70641

**Published:** 2025-05-21

**Authors:** Sirirat Anutrakulchai, Sajja Tatiyanupanwong, Sarassawan Kananuraks, Eakalak Lukkanalikitkul, Sawinee Kongpetch, Wijittra Chotmongkol, Michael G Morley, Wilaiphorn Thinkhamrop, Bandit Thinkhamrop, Chadarat Kleebchaiyaphum, Krongsin Khianchanach, Theenatchar Chunghom, Katharine E Morley

**Affiliations:** 1 Division of Nephrology Faculty of Medicine Khon Kaen University Khon Kaen Thailand; 2 Kidney Unit Department of Internal Medicine Chaiyaphum Hospital Chaiyaphum Thailand; 3 Division of Nephrology Department of Medicine Khon Kaen Hospital Khon Kaen Thailand; 4 Center of Excellence in Kidney Diseases, Srinagarind Hospital Faculty of Medicine Khon Kaen University Khon Kaen Thailand; 5 Massachusetts Eye and Ear Infirmary Harvard Ophthalmology AI Lab Harvard Medical School Boston, MA United States; 6 Department of Mathematics and Statistics Faculty of Medicine Bangkok Thonburi University Bangkok Thailand; 7 Data Management and Statistical Analysis Center Faculty of Public Health Khon Kaen University Khon Kaen Thailand; 8 Kidney Unit, Srinagarind Hospital Faculty of Medicine Khon Kaen University Khon Kaen Thailand; 9 Harvard Medical School Center for Global Health Massachusetts General Hospital Boston, MA United States

**Keywords:** overhydration, peritoneal dialysis, smartphone app, mobile health, chronic kidney disease, hospitalization

## Abstract

**Background:**

Overhydration is associated with increased morbidity and mortality in patients on peritoneal dialysis (PD). Early detection of overhydration is possible by monitoring hydration metrics, but the critical gap for treatment is obtaining timely and actionable data.

**Objective:**

This study compares the detection of overhydration and clinical outcomes in patients on PD using the Chronic Kidney Disease—Peritoneal Dialysis (CKD-PD) smartphone app with standard monitoring and management.

**Methods:**

An open-label randomized controlled trial was conducted at 3 hospitals in northeast Thailand. Enrolled participants from PD clinics were randomized into 2 equal groups: CKD-PD (App users) and usual management (No-App). Participants or their caregivers in the App group recorded hydration metrics in the CKD-PD app, which were uploaded to a central database monitored by nephrology staff. The No-App group used a handwritten logbook. Both groups had bimonthly clinic visits. The primary outcome was the incidence rate ratio (IRR) for clinical interventions for overhydration. Secondary outcomes included hospitalizations, technique failure, and death.

**Results:**

A total of 208 participants were randomized into App (N=103) and No-App (N=105) groups with the median follow-up time of 11.2 months. Hydration metric upload compliance in the App group was 85.7% (IQR 71.4-95.6). The IRR of overall interventions for overhydration was 2.51 times higher in the App group (95% CI 2.18-2.89; *P*<.001). Types of clinical interventions for overhydration differed between groups with dietary change and prescription of antihypertensive drugs more frequent in App users and diuretics and change of dialysis prescription more frequent in the No-App group. Hospitalizations were significantly higher in the No-App group due to any cause (adjusted IRR 1.58) and volume overload (adjusted IRR 4.07). There was no significant difference in survival analysis and technique failure between the 2 groups.

**Conclusions:**

Use of the CKD-PD app improved early detection of overhydration and early treatment interventions, resulting in fewer all-cause and volume overload hospitalizations.

**Trial Registration:**

ClinicalTrials.gov NCT04797195; https://clinicaltrials.gov/study/NCT04797195

## Introduction

The global prevalence of chronic kidney disease (CKD) is 11%-13% [1] and increasing in low and middle-income countries paralleling the rising rates of diabetes and hypertension and lack of access to early CKD treatment [2-4]. The use of peritoneal dialysis (PD), a modality of home renal replacement therapy for patients with kidney failure (KF), is growing to meet this demand, especially in Asian countries with limited resources [5-7]. In 2008, Thailand adopted a “PD first” policy to offer this benefit in its Universal Health Coverage Scheme [8], resulting in an 11.3-fold increase in patients on PD from 2168 to 24,439 cases from 2008 to 2022 [9].

Overhydration (OH) is one of the most common complications in patients on PD. Clinical manifestations of OH include peripheral edema, dyspnea, and hypertension [10]. It is estimated that 50%-60% of patients on PD are overhydrated, with severe OH producing clinical symptoms in 25% [11,12]. OH is associated with increased morbidity and mortality due to infections, including peritonitis [13], and major adverse cardiovascular events such as myocardial infarction, pulmonary edema, stroke, and hypertensive crisis [10]. OH alone, independent of coexisting cardiovascular pathologies, is a risk factor for increased morbidity and mortality [14]. Early detection of OH is possible by monitoring patients’ hydration metrics. In northeast Thailand, nephrologists and PD nurses routinely assess hydration status of patients on PD by reviewing hydration metrics—blood pressure (BP), body weight (BW), and ultrafiltration (UF) volume collected and recorded by patients in a handwritten logbook during scheduled bimonthly clinic visits. The critical gap for the treatment of OH lies in obtaining these hydration metrics in a timely and actionable format when early interventions can be made to improve quality of life, decrease health care costs, and reduce morbidity and mortality [15].

Our multidisciplinary team developed a smartphone app (“Chronic Kidney Disease—Peritoneal Dialysis” [CKD-PD] app) for Thai patients on PD [16]. Hydration metrics are recorded in the CKD-PD app and uploaded to a central health database merging patient-collected data with hospital and clinic records [17]. The objective of this study was to compare the detection of OH and clinical outcomes in patients on PD using the CKD-PD app with the standard monitoring and management of patients on PD.

## Methods

### Study Design and Participants

This study was an open-label randomized controlled trial (RCT) conducted between December 2021 and February 2023 at the three hospitals in northeast Thailand: (1) Srinagarind Hospital (academic tertiary), (2) Khon Kaen Hospital (urban provincial), and (3) Chaiyaphum Hospital (rural provincial). Patients with KF currently on PD at clinics of these hospitals were enrolled using the inclusion criteria: willingness to participate, aged 18 years and older, access to a smartphone, and the ability to use the CKD-PD app (independently or with a surrogate). Randomization codes for the 2 groups were generated by the statistician using computer software. Block randomization with varying block sizes of 2 and 4 was used to ensure allocation concealment and balance between the arms. The randomization sequence was created using a random number generator and was implemented without stratification. The allocation sequence was concealed from study personnel and participants until assignment when research nurses enrolled and allocated participants. After writing informed consent, all participants were randomized by software (Sealed Envelope Ltd, 2024. Create a Blocked Randomization List [available online]) into 2 equal groups at each hospital, 1 using the CKD-PD app (“App” group) and 1 receiving usual management (“No-App” group). A CONSORT-EHEALTH (Consolidated Standards of Reporting Trials of Electronic and Mobile Health Applications and Online Telehealth; version 1.6.1) checklist was completed (Multimedia Appendix 1).

### Ethical Considerations

The study protocol was approved by the Khon Kaen University ethics committee for human research (HE 621494) in accordance with the ethical principles of the Declaration of Helsinki, the Good Clinical Practice guidelines. It was registered in the Clinical Trials Registry (ClinicalTrials.gov ID#NCT04797195 with the first postdate March 15, 2021) prior to patient enrollment. Ethics approval was obtained from the Massachusetts General Brigham institutional review board (protocol 2019P002648). All participants were informed that their participation is voluntary, and they could drop out of the study at any time without impact on their treatment. The original informed consent allows the secondary data analysis without additional consent. Signed copies of the informed consent were stored with study records, and a copy was given to the participants. All study data were securely stored in password-protected computers and participant information was deidentified by using a unique identification code. Participants were provided with home hydration metric–monitoring equipment, including BW scale, hanging scale, and BP measurement device. Compensation was provided for travel to and from PD clinic visits.

### Description of the CKD-PD App

#### Overview

The CKD-PD app was developed at Khon Kaen University for individuals who want to track their own renal status by monitoring BP, BW, blood sugar levels, and water intake and output. It is available for free download in both Android and iOS formats. Patients on PD can enter PD metrics such as UF volume and set up a secure account with a username and password to send their PD data recorded at home to the Chronic Kidney Disease Prevention in the Northeast of Thailand (CKDNET) database in the Thai Care Cloud [16,17] for monitoring by their PD care providers. The key features of the CKD-PD app are (1) daily data entry for continuous monitoring of hydration metrics, (2) graphical display of hydration status for app users, (3) near real-time data for monitoring patients’ hydration metrics by PD clinic staff, (4) an integrated secure chat function (LINE professional) to facilitate communication between patients on PD and clinic staff, and (5) integration of hydration metrics from the CKD-PD app with electronic health data in the CKDNET database on the Thai Care Cloud. The CKD-PD app (Multimedia Appendix 2) underwent usability testing and iterative improvements prior to the study [16].

#### Study Protocol, Intervention, and Definition of Exposures

All participants continued standard bimonthly PD clinic visits during the study period. The study participation period was 12 months, with the actual duration varying by enrollment date, last scheduled follow-up date, or premature study termination. Participants were assigned a PD clinic visit appointment date based on their randomization allocation to reduce contamination between the App group and No-App group attending PD clinics on the same day. Participants in both groups used home BW scales and hanging scales to weight dialysate bags to calculate UF volume and were provided with a new automatic arm BP monitor (Omron).

#### Hydration Metric Collection

Hydration metric collection in both groups was performed at home by participants or their surrogate daily and included (1) morning BP, (2) BW before first dialysis cycle, and (3) UF volume: difference in total weight of the dialysate fluid bags before and after each peritoneal dwell period for the preceding 24 hours. The No-App group received usual care: (1) “PD logbook”—a handwritten logbook to record hydration metrics, (2) bimonthly PD clinic appointments, and (3) instructions to contact the clinic for any concerns. No other outreach was conducted unless the participant contacted the PD staff or sought care at the PD clinic or emergency department. The App group received training on how to use the CKD-PD app including hydration metric entry, self-monitoring, and in-app communication features. Participants were instructed to record their hydration metrics data daily using the app. The PD clinic staff checked participant hydration metric data in the CKDNET database weekly. If the hydration metrics were not uploaded, the PD staff reminded the participant by telephone or chat app. Participants were provided cellular access in cases of issues with web instability or access so that they could temporarily check their hydration metrics via LINE professional mobile communication app. App group participants also had bimonthly PD clinic appointments and received instructions to contact the clinic for any concerns.

#### Hydration Metric Monitoring

PD clinic staff monitored hydration metrics from the App group weekly using the CKDNET database and during scheduled bimonthly clinic visits and unscheduled clinic contacts in the App group. Hydration metrics from the No-App group were monitored by PD clinic staff during scheduled bimonthly clinic visits and unscheduled clinic contacts. The baseline dry weight was individually set using the bioimpedance device (Body Composition Monitor; Fresenius Medical Care), together with clinical adjustments by nephrologists. Subsequently, the dry weight was periodically adjusted based on clinical assessments; bioimpedance measurements were performed if there was uncertainty. Hydration metrics were classified as normal, need monitoring, and action required. Criteria for an action-required alert were one or more of the following parameters: (1) deviation in current BW of >3% from set dry weight, (2) BP >140/90 mm Hg, and (3) UF volume of <500 mL in patients with anuresis. In the event of an action-required alert, the PD nurse initiated contact with the participant to review hydration metrics and symptoms. If the PD nurse confirmed abnormal hydration metrics or clinical symptoms consistent with OH, a critical alert was given, and the case was referred to a study nephrologist for review and clinical intervention if indicated. Hydration metrics were classified as need monitoring if trending in an abnormal direction but did not fulfill the action-required criteria.

#### Critical Alerts

Critical alerts were made if any of the following events occurred: (1) abnormal hydration metrics or clinical symptoms consistent with OH on review of hydration metrics by PD staff, (2) abnormal hydration metrics or clinical symptoms consist with OH during scheduled PD clinic visit, (3) participant reported abnormal hydration metrics or symptoms of OH verified by PD staff, and (4) emergency or unscheduled hospital visit. In the event of a critical alert, a critical alert form was initiated and the following data collected: source of critical alert, clinical symptoms, hydration metric abnormalities, type of clinical intervention if indicated, and hospital referral, if required.

#### Clinical Interventions for OH

In the No-App group, clinical interventions for OH were made at an unscheduled contact (eg, outreach to PD clinic or emergency visit) or scheduled contact (routine clinic visit every 2 months). In the App group, clinical interventions were made when PD clinic staff contacted the patient in response to an action-required alert, in addition to unscheduled and scheduled PD clinic contact. An episode and type of clinical intervention decided by nephrologists was defined as a treatment intervention for abnormal hydration metrics or clinical symptoms of OH and included changes in antihypertension medications, diuretic medications, or both; fluid and salt restriction; modification of dialysis prescription; changes of PD solution such as hypertonic dextrose solution or icodextrin solution; or referral for urgent visit.

### Data Collection

Baseline data were collected from medical records and patient interviews. Hydration metrics, hydration metric monitoring, clinical symptoms, PD clinic contact, and clinical interventions for OH were recorded in real time using study logs and an electronic data capture system. Outcomes of hospitalization, death, and technique failure were collected at the time of event notification and from the hospital information system.

### Statistical Analysis

Sample size was calculated to determine the primary outcome by using a difference in incidence rates (IRs) between 2 Poisson means with 25% precision. Preliminary data from Srinagarind Hospital indicated the intervention rate for OH to be 4 times per week in 91 patients or a mean intervention rate of 2.3 times per patient-year. Assuming a 2-fold increase in the mean event rate for patients using the CKD-PD app or 4.6 times per patient-year in the CKD-PD app group, the desired total sample size was 80 patients for each group, using a 2-sided, large-samples *z* test of the Poisson event-rate difference at a significance level of .05. Allowing for a 10% dropout rate, a total of at least 200 patients were recruited. Recruitment targets for each facility were determined by the number of patients followed in the PD clinic: Srinagarind Hospital: 20%, Khon Kaen Hospital: 30%, and Chaiyaphum Hospital: 50%.

Baseline characteristics were described by summary statistics. Percent distribution was presented for all categorical variables. Mean (SD) and median (IQR) were presented for continuous variables. The outcomes were analyzed as intent-to-treat. The primary outcome was presented as IR of interventions for OH and the incidence rate ratio (IRR) and its 95% CI were estimated to compare both groups. The generalized estimating equation with baseline values adjustment was performed to compare the repeated measures between the App and No-App groups. Secondary outcome for hospitalization was assessed using a multivariate Poisson regression analysis for IRR adjusted with factors that accounted for a *P* value of <.05 in univariate analysis, a significant difference in baseline characteristics between the 2 groups, and hospital levels. Survival analysis was compared between the groups. All statistical tests were 2-sided and performed using STATA (version 17; StataCorp), using a significance level of .05.

## Results

### Baseline Characteristics and Demographic Data

A total of 224 patients were eligible for the study with 16 cases being excluded for (1) failure to meet the inclusion criteria (n=11) and (2) declining to participate (n=5). A total of 208 participants were randomized into the 2 groups at each study site resulting in 103 subjects in the App group and 105 cases in the No-App group (Table 1). The study population was 43.8% (91/208) male participants, with a mean age 54.3 (SD 15.0) years, and median duration of PD treatment of 19.0 (IQR 7.6-35.4) months. Most participants had an education level below high school (128/208, 61.5% of participants) and lived a median distance from PD clinic of 41 km (IQR 15-80). Diabetic nephropathy and hypertensive nephrosclerosis were the 2 major causes of KF. Both groups had similar clinical characteristics except significantly higher levels of BP, serum calcium, serum parathyroid hormone, peritonitis episodes, and less use of erythropoietin-stimulating agent in the App group. Multimedia Appendix 3 demonstrates baseline characteristics of participants treated at the 3 hospitals. Participants from Srinagarind Hospital were older, had higher education levels, and higher percentages of smoking, diabetes, and cardiovascular comorbidities. Clinical signs of OH and medication use also varied between sites with more controlled hypertension with antihypertensive medication and lesser use of erythropoietin-stimulating agent at Srinagarind Hospital compared with Chaiyaphum Hospital, but more pulmonary congestion and edema were documented at Srinagarind Hospital. There were significant variations in some PD techniques, modes, and history of PD-related complications between the hospitals.

**Table 1:** Baseline characteristics of participants.

Characteristics						Total (n=208)		App group (n=103)					No-App group (n=105)					*P* value
Age (years), mean (SD)						54.3 (15.0)		53.9 (15.5)					54.8 (14.5)					.66
Male sex, n (%)						91 (43.8)		40 (38.8)					51 (48.6)					.16
**Educational levels, n (%)**																		.10
	Less than high school				128 (61.5)		57 (55.3)					71 (67.6)						
	High school				47 (22.6)		27 (26.2)					20 (19.0)						
	Occupational program				9 (4.3)		5 (4.9)					4 (3.8)						
	Bachelor’s degree				19 (9.1)		9 (8.7)					10 (9.5)						
	More than bachelor’s degree				5 (2.4)		5 (4.9)					0 (0)						
Distance from PD^a^ clinic (km), median (IQR)						41 (15-80)		40.5 (15-86)					42 (20-77)					.93
**Cause of CKD^b^, n (%)**																		.40
	Diabetes nephropathy				96 (46.2)		41 (39.8)					55 (52.4)						
	Hypertensive nephrosclerosis				47 (22.6)		23 (22.3)					24 (22.9)						
	Glomerulonephritis				11 (5.3)		7 (6.8)					4 (3.8)						
	Gouty nephropathy				4 (1.9)		2 (1.9)					2 (1.9)						
	Others				11 (5.3)		6 (5.8)					5 (4.8)						
	Unknown				39 (18.8)		24 (23.3)					15 (14.3)						
**Comorbidities, n (%)**																		.56
	None				12 (5.8)		7 (6.8)					5 (4.8)						
	Hypertension				155 (74.5)		78 (75.7)					77 (73.3)						
	Dyslipidemia				41 (19.7)		20 (19.4)					21 (20.0)						
	Coronary heart disease				11 (5.3)		3 (2.9)					8 (7.6)						
	Cerebrovascular disease				8 (3.8)		5 (4.9)					3 (2.9)						
	Others				45 (21.6)		20 (19.4)					25 (23.8)						
**Smoking history, %**																		.20
	Never/former/current				73.1/25.5/1.4		78.6/20.4/1.0					67.6/30.5/1.9						
**Clinical parameters**																		
	Body weight (kg), mean (SD)				58.7 (13.2)					58.2 (11.8)					59.3 (14.4)		.55	
	BMI (kg/m²), mean (SD)				22.8 (4.1)		22.6 (3.3)					23.0 (4.7)					.50	
	SBP^c^ (mm Hg), mean (SD)				148.2 (26.1)		152.5 (27.0)					144.0 (24.6)					.02	
	DBP^d^ (mm Hg), mean (SD)			81.1 (18.8)							83.7 (19.9)					78.6 (17.4)		.05
	Daily urine (mL/day), median (IQR)				300 (50-664)		270 (33-560)					400 (100-700)					.11	
**Clinical signs of overhydration, n (%)**																		
		Orthopnea			2 (1.0)		0 (0)					2 (1.9)					.16	
		PND^e^/DOE^f^			2 (1.0)		0 (0)					2 (1.9)					.16	
		Hypertension (BP^g^ >140/90 mm Hg)			65 (31.3)		35 (34.0)					30 (28.6)					.40	
		Edema			47 (22.6)		19 (18.4)					28 (26.7)					.16	
**Medications, n (%)**																		
	Furosemide				123 (59.1)				60 (58.3)					63 (60.0)			.98	
	Antihypertensive				182 (87.5)		91 (88.3)					91 (86.7)					.71	
	ESA^h^				190 (91.3)		90 (87.4)					100 (95.2)					.04	
**Laboratory profiles**																		
	Hemoglobin (g/dL), mean (SD)				9.9 (1.9)		10.0 (1.9)					9.8 (1.9)					.52	
	Fasting blood sugar (mg/dL)^i^, mean (SD)				131.8 (60.3)		138.1 (64.8)					126.7 (56.3)					.27	
	Blood urea nitrogen (mg/dL), mean (SD)				53.3 (19.2)		54.1 (18.1)					52.6 (20.2)					.58	
	Creatinine (mg/dL), mean (SD)				10.3 (4.4)		10.6 (4.4)					10.1 (4.4)					.43	
	Sodium (mEq/L), mean (SD)				135.5 (5.2)		135.6 (4.9)					135.4 (5.4)					.86	
	Potassium (mEq/L), mean (SD)				4.1 (0.8)		4.1 (0.7)					4.0 (0.8)					.34	
	Calcium (mg/dL), mean (SD)				8.4 (1.0)		8.6 (0.9)					8.3 (1.1)					.049	
	Phosphorus (mg/dL), mean (SD)				4.5 (1.8)		4.5 (1.9)					4.5 (1.8)					.91	
	Albumin (g/dL), mean (SD)				3.3 (0.6)		3.3 (0.6)					3.3 (0.6)					.51	
	Parathyroid hormone (pg/mL), median (IQR)				279 (153-473)		324 (155-596)					234 (139-396)					.01	
**PD information**																		
	Duration of PD (months), median (IQR)				19.0 (7.6-35.4)		22.3 (10.2-43.8)					15.8 (6.2-32.2)					.05	
	**Mode of PD, %**																.25	
			CAPD^j^/DAPD^k^		52.4/33.7		52.4/35.0					52.4/32.4						
			NIPD^l^/CCPD^m^		12.5/1.4		9.7/2.9					15.2/0.0						
	**Types of peritoneal membrane transport, %**																.18	
			Fast/average/slow		6.3/85.6/8.2		2.9/91.3/5.8					8.6/81.9/9.5						
	**PD exchanger, %**																.12	
			Patient/family member		54.8/45.2		60.2/39.8					49.5/50.5						
	**PD solution, n (%)**																	
			1.5% dextrose		206 (99.0)		102 (99.0)					104 (99.0)					>.99	
			2.5% dextrose		19 (9.1)		11 (10.7)					8 (7.6)					.44	
			4.25% dextrose		16 (7.7)		4 (3.9)					12 (11.4)					.07	
			7.5% icodextrin		2 (1.0)		1 (1.0)					1 (1.0)					>.99	
	Daily UF^n^ (mL/day), median (IQR)				700 (450-1000)		700 (414-943)					700 (500-1000)					.61	
	History of peritonitis, n (%)				46 (22.1)		30 (29.1)					16 (15.2)					.02	
	History of exit site infection, n (%)				30 (14.4)		17 (16.5)					13 (12.4)					.40	
	History of catheter malfunction, n (%)				14 (6.7)		5 (4.9)					9 (8.6)					.29	

^a^PD: peritoneal dialysis.

^b^CKD: chronic kidney disease.

^c^SBP: systolic blood pressure.

^d^DBP: diastolic blood pressure.

^e^PND, paroxysmal nocturnal dyspnea.

^f^DOE, dyspnea on exertion.

^g^BP: blood pressure.

^h^ESA: erythropoiesis-stimulating agent.

^i^Tested in patients with diabetes.

^j^CAPD: continuous ambulatory peritoneal dialysis.

^k^DAPD: daytime ambulatory peritoneal dialysis.

^l^NIPD: nocturnal intermittent peritoneal dialysis.

^m^CCPD: continuous cycling peritoneal dialysis.

^n^UF: ultrafiltration.

**Figure 1:**
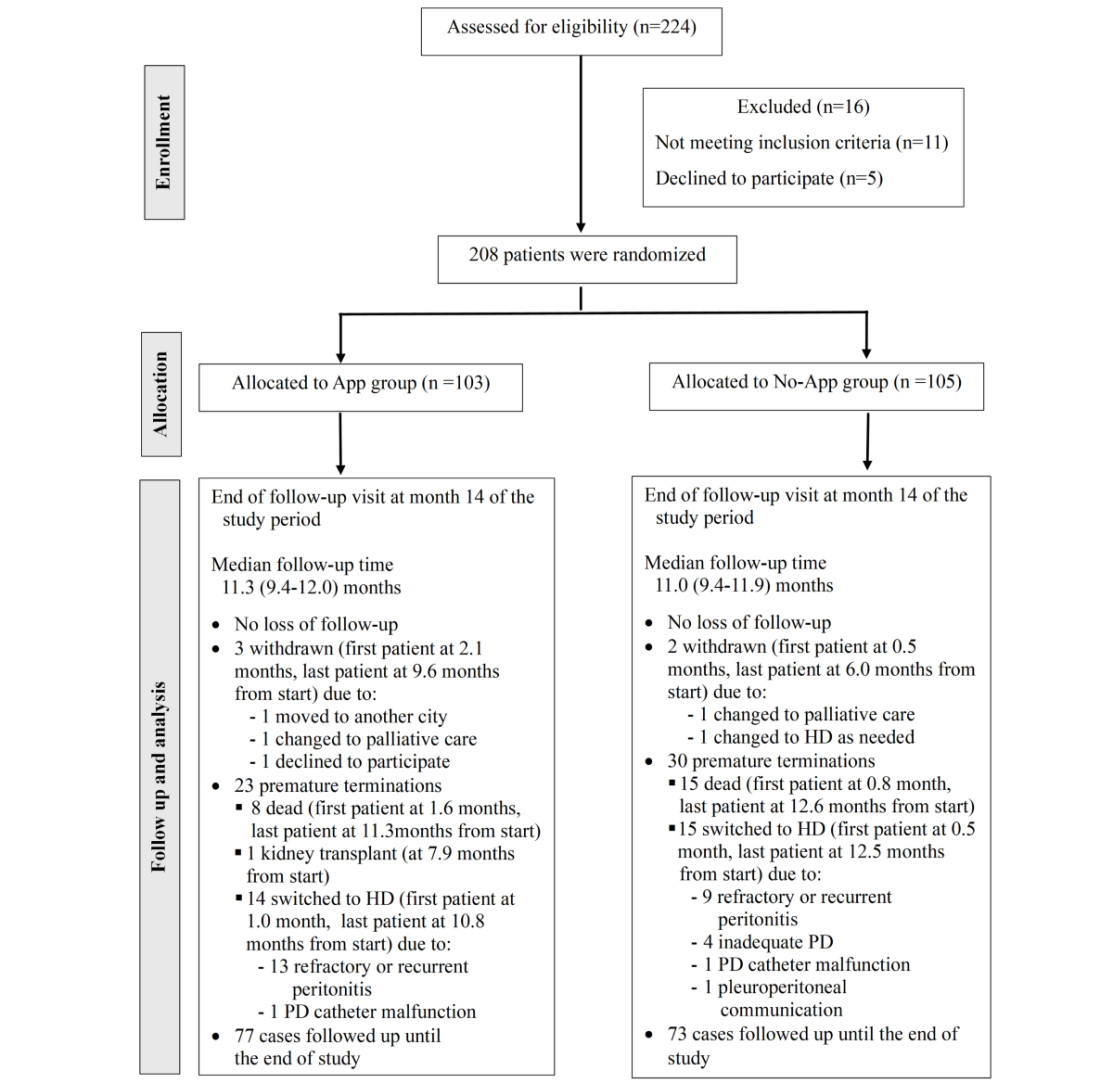
Scheme of the study flow. HD: hemodialysis; PD: peritoneal dialysis.

There were no participants lost to follow-up during the study period. Median follow-up times were similar between the App (11.2, IQR 9.4-12.0 months) and No-App groups (11.0, IQR 9.4-11.9 months; *P*=.49). Study withdrawal occurred in 3 App group and 2 No-App group participants. There were 23 participants who were prematurely terminated in the App group and 30 in the No-app group. At the end of study, 74.8% (77/103) of participants in the App group and 69.5% (73/105) of participants in the No-App group completed the entire follow-up period as shown in the study flow diagram (Figure 1).

### Hydration Metric Monitoring and PD Clinic Communication

Table 2 shows the results of hydration metric monitoring and communication with the PD clinic. Median percentage of hydration metric upload compliance in the App group was 85.7% (IQR 71.4-95.6), with the highest rate by participants at Khon Kaen Hospital (91.8%, IQR 76.9-99.4). Total follow-up visits in the App group were 3657, with 3145 using the CKD-PD app and 512 at scheduled PD clinic appointment. The No-App group had 475 follow-up visits, all at scheduled PD clinic appointments. Critical alerts for OH were significantly greater in the App group (n=473) than in the No-App group (n=164; *P*<.001), with 74.8% (354/473) of participants having OH alerts resulting from review of uploaded hydration metrics from the CKD-PD app. There were 118 critical alerts for OH in the App group compared with 161 in the No-App group at scheduled PD clinic visits. In the App group, 1 participant had a critical alert for OH after an emergency unit visit compared with 3 in the No-App group, both significant (*P*<.001). The most frequent mode of contact at all hospitals between PD clinic staff and participants with abnormal hydration metrics and OH in the App group was telephone, followed by chat app.

**Table 2:** Hydration metric monitoring and peritoneal dialysis clinic communication.

		All sites		Srinagarind			Khon Kaen			Chaiyaphum	
		App (n=103)	No-App (n=105)	App (n=20)	No-App (n=20)	App (n=35)		No-App (n=37)	App (n=48)		No-App (n=48)
Median % hydration metrics uploaded from participants, median (IQR) percentage of compliance		85.7 (71.4-95.6)	N/A^a^	82.8 (74.2-90.1)	N/A	91.8 (76.9-99.4)		N/A	81.7 (61.2-94.5)		N/A
**Total follow-up visits, n**		3657	475	923	88	1555		192	1178		195
	App visits	3145	N/A	823	N/A	1352		N/A	970		N/A
	Scheduled PD^b^ clinic visit	512	475	100	88	204		192	208		195
**Total critical alerts for overhydration, n**		473	164^c^	196	38^c^	189		97^c^	88		29^c^
	From uploaded hydration metrics	354	N/A	157	N/A	141		N/A	56		N/A
	From PD clinic visit and emergency visits	119	164^c^	39	38	48		97	32		29
**Method of overhydration critical alert, n (%)**		473 (100)	164 (100)^c^	196 (100)	38 (100)^c^	189 (100)		97 (100)^c^	88 (100)		29 (100)^c^
	Scheduled PD clinic visit	118 (24.9)	161 (98.2)	39 (19.9)	36 (94.7)	48 (25.4)		97 (100)	31 (35.2)		28 (96.6)
	Participant hydration metric review	9 (1.9)	0 (0)	5 (2.6)	0 (0)	2 (1.1)		0 (0)	2 (2.3)		0 (0)
	PD staff hydration metric review	345 (72.9)	0 (0)	152 (77.6)	0 (0)	139 (73.5)		0 (0)	54 (61.4)		0 (0)
	Emergency department visit	1 (0.2)	3 (1.8)	0 (0)	2 (5.3)	0 (0)		0 (0)	1 (1.1)		1 (3.4)
**Mode of contact for overhydration, n (%)**		473 (100)	164 (100)^c^	196 (100)	38 (100)^c^	189 (100)		97 (100)^c^	88 (100)		29 (100)^c^
	Hospital visits^d^	120 (25.4)	163 (99.4)	38 (19.4)	38 (100)	51 (27.0)		97 (100)	31 (35.2)		28 (96.6)
	Telephone	235 (49.7)	1 (0.6)	158 (80.6)	0 (0)	23 (12.2)		0 (0)	54 (61.4)		1 (3.4)
	Chat app	118 (24.9)	0 (0)	0 (0)	0 (0)	115 (60.9)		0 (0)	3 (3.4)		0 (0)

^a^N/A: not applicable.

^b^PD: peritoneal dialysis.

^c^*P* value <.001 between the App group and the No-App group.

^d^Hospital visits included PD clinic visits and emergency visits.

### Critical Alerts and Clinical Interventions for OH

Clinical signs and symptoms resulting in critical alerts for OH are shown in Table 3, which demonstrates that the total numbers of critical alerts and clinical interventions for OH were significantly higher in the App group (473 vs 164 episodes; *P*<.001). The percentage of critical alerts due to increased BW was higher in the App group (200/473, 42.3% of participants vs 48/164, 29.3% of participants; *P*=.003), but clinical signs of OH were significantly higher in the No-App group for edema (116/164, 70.7% of participants vs 154/473, 32.6% of participants; *P*<.001) and for respiratory symptoms (10/164, 6.1% of participants vs 5/473, 1.1% of participants; *P*<.001) predominately noticed at Srinagarind Hospital (all *P*<.001). The No-App groups significantly presented more edema at Khon Kaen Hospital (63/97, 65.0% of participants vs 33/189, 17.5% of participants; *P*<.001) and more uncontrolled hypertension at Chaiyaphum Hospital (15/29, 51.7% of participants vs 27/88, 30.7% of participants; *P*=.04). There was significant difference in the types of clinical interventions for OH between the App and No-App groups with higher percentages of dietary restrictions (*P*=.03) and antihypertensive drug adjustments (*P*=.01) in the App group, and of advanced OH treatment (diuretics [*P*=.001], PD hypertonic solution [*P*<.001], and change of PD prescription [*P*=.003]) in the No-App group.

**Table 3:** Overhydration critical alert signs and symptoms and clinical interventions for overhydration.

		All sites			Srinagarind			Khon Kaen			Chaiyaphum	
		App (n=103)	No-App (n=105)	App (n=20)		No-App (n=20)	App (n=35)		No-App (n=37)	App (n=48)		No-App (n=48)
**Clinical signs and symptoms of overhydration resulting in critical alerts, n (%)**												
	Total critical alerts for overhydration	473 (100)	164 (100)	196 (100)		38 (100)	189 100)		97 (100)	88 (100)		29 (100)
	High blood pressure	128 (27.1)	50 (30.5)	58 (29.6)		9 (23.7)	4 (22.8)		26 (26.8)	27 (30.7)		15 (51.7)^a^
	Weight gain of >3% from target weight	200 (42.3)	48 (29.3)^a^	104 (53.1)		9 (23.7)^a^	72 (38.1)		28 (28.9)	24 (27.3)		11 (37.9)
	Edema	154 (32.6)	116 (70.7)^a^	79 (40.3)		35 (92.1)^a^	33 (17.5)		63 (65.0)^a^	42 (47.7)		18 (62.1)
	Dyspnea, orthopnea and PND^b^	5 (1.1)	10 (6.1)^a^	4 (2.0)		8 (21.1)^a^	0 (0)		0 (0)	1 (1.1)		2 (6.9)
	Abdominal pain	0 (0)	1 (0.6)	0 (0)		1 (2.6)	0 (0)		0 (0)	0 (0)		0 (0)
	Fever	0 (0)	1 (0.6)	0 (0)		1 (2.6)	0 (0)		0 (0)	0 (0)		0 (0)
	Cloudy dialysate effluent	1 (0.2)	0 (0)	1 (0.5)		0 (0)	0 (0)		0 (0)	0 (0)		0 (0)
	Mechanical problem	1 (0.2)	0 (0)	1 (0.5)		0 (0)	0 (0)		0 (0)	0 (0)		0 (0)
	Other symptoms	6 (1.3)	5 (3.0)	6 (4.6)		3 (7.9)	0 (0)		2 (2.1)	0 (0)		0 (0)
**Clinical interventions for overhydration from critical alert, n (%)**												
	Total critical alerts for overhydration	473 (100)	164 (100)	196 (100)		38 (100)	189 (100)		97 (100)	88 (100)		29 (100)
	Restrict oral fluid and salt intake	436 (92.2)	142 (86.6)^a^	178 (90.8)		35 (92.1)	186 (98.4)		94 (96.9)	72 (81.8)		13 (44.8)^a^
	Anti-HT^c^ drugs	110 (23.3)	23 (14.0)^a^	97 (49.5)		16 (42.1)	2 (1.1)		1 (1.0)	11 (12.5)		6 (20.7)
	Diuretic drugs	32 (6.8)	25 (15.2)^a^	23 (11.7)		16 (42.1)^a^	8 (4.2)		9 (9.3)	1 (1.1)		0 (0)
	Hypertonic dialysis solution	79 (16.7)	55 (33.5)^a^	42 (21.4)		10 (26.3)	29 (15.3)		40 (41.2)^a^	8 (9.1)		5 (17.2)
	Change of PD^d^ prescription	35 (7.4)	25 (15.2)^a^	17 (8.7)		6 (15.8)	4 (2.1)		12 (12.4)^a^	14 (15.9)		7 (24.1)
	7.5% icodextrin PD solution	4 (0.8)	0 (0)	4 (2.0)		0 (0)	0 (0)		0 (0)	0 (0)		0 (0)

^a^*P* value of <.05 between the App and No-App groups.

^b^PND: paroxysmal nocturnal dyspnea.

^c^Anti-HT drug: antihypertensive drug.

^d^PD: peritoneal dialysis.

Clinical parameters of repeated measures of hydration metrics recorded throughout the study time were compared between the App and No-App groups by using the generalized estimating equation with baseline values adjustment as shown in Table 4. Overall results showed that better BP control (*P*<.001) and greater reduction of weight gain in the App group (*P*<.001) by mean differences of systolic blood pressure were –8.6 (–12.0 to –5.1 mm Hg; *P*<.001), and BW –0.9 (–1.7 to –0.1 kg; *P*=.028) between the groups.

**Table 4:** Hydration metric monitoring and clinical signs and symptoms recorded during the study period.

			All sites				Srinagarind				Khon Kaen				Chaiyaphum		
			App (n=103)		No-App (n=105)		App (n=20)		No-App (n=20)		App (n=35)		No-App (n=37)		App (n=48)		No-App (n=48)
**Total follow-up visits, n**			3657		475		923		88		1555		192		1178		195
	App visits	3145		N/A^a^		823		N/A		1352		N/A		970		N/A	
	Scheduled PD^b^ clinic visit	512		475		100		88		204		192		208		195	
**Clinical parameters recorded during the study period**																	
	UF^c^ volume^d^ (mL/day), median (IQR)	800 (500-1000)		800 (500-1074)		700 (437-1000)		600 (400-900)		800 (611-1000)		800 (600-1100)		700 (400-1000)		700 (500-1078)	
	Change of BW^e,f^ from baseline (kg), mean (SD)	–0.74 (3.05)		–0.08 (4.03)^g^		–0.77 (2.53)		–1.07 (2.55)		–0.29 (3.26)		0.16 (4.66)		–1.26 (3.04)		0.12 (3.84)^g^	
	SBP^h,i^ (mm Hg), mean (SD)	133 (19)		141 (24)^g^		134 (18)		137 (20)		128 (16)		142 (23)^g^		140 (20)		143 (26)^g^	
	DBP^j,k^ (mm Hg), mean (SD)	79 (14)		77 (16)^g^		77 (15)		67 (13)		78 (11)		79 (15)		83 (14)		79 (16)^g^	
	Uncontrolled BP^l^ (>140/90 mm Hg), times (%)	372/3421 (10.9)		83/474^g^ (17.5)		95/867 (11.0)		9/87 (10.3)		85/1434 (5.9)		38/192^g^ (19.8)		192/1120 (17.1)		44/195 (22.6)	

^a^N/A: not applicable.

^b^PD: peritoneal dialysis.

^c^UF: ultrafiltration.

^d^Mean difference in ultrafiltration rate was 20.54 (95% CI –61 to 102) mL/day for all sites (*P*=.62), –15.01 (95% CI –160 to 130) mL/day for Srinagarind (*P*=.84), –31.95 (95% CI –111 to 47) mL/day for Khon Kaen (*P*=.43), and 72.93 (95% CI –92 to 237) mL/day for Chaiyaphum (*P*=.39).

^e^BW: body weight.

^f^Mean difference in body weight reduction was –0.92 (95% CI –1.73 to 0.10) kg for all sites (*P*=.03), 0.50 (95% CI –0.70 to 1.70) kg for Srinagarind (*P*=.42), –0.66 (95% CI –2.27 to 0.95) kg for Khon Kaen (*P*=.42), and –1.65 (95% CI –2.81 to 0.49) kg for Chaiyaphum (*P*=.005).

^g^*P* value of <.05 between the App and Non-app groups.

^h^SBP: systolic blood pressure.

^i^Mean difference in systolic blood pressure was –8.55 (95% CI –12.01 to –5.09) mm Hg for all sites (*P*<.001), –5.94 (95% CI –12.98 to 1.11) mm Hg for Srinagarind (*P*=.10), –11.15 (95% CI –16.84 to –5.47) mm Hg for Khon Kaen (*P*<.001), and –7.15 (95% CI –12.66 to 1.64) mm Hg for Chaiyaphum (*P*=.01).

^j^DBP: diastolic blood pressure.

^k^Mean difference in diastolic blood pressure was 1.31 (95% CI –0.99 to 3.61) mm Hg for all sites (*P*=.27), 7.55 (95% CI 2.84 to 12.27) mm Hg for Srinagarind (*P*=.002), –0.49 (95% CI –3.75 to 2.77) mm Hg for Khon Kaen (*P*=.77), and 0.16 (95% CI –3.68 to 4.01) mm Hg for Chaiyaphum (*P*=.93).

^l^BP: blood pressure.

### Primary Outcome of IRR for OH Interventions

The primary outcome of the IR of clinical interventions for OH and comparison of IRR between the App and No-App groups are reported in Table 5. The person-times were 1037.84 months in the App group and 1009.64 months in the No-App group. The IR of overall clinical intervention was 2.5 times significantly higher in the App group, that is, IRR 0.4 (95% CI 0.35-0.46; *P*<.001) using the App group as reference. Analysis of specific clinical intervention types showed that dietary restriction and prescription of antihypertensive drugs were significantly more frequent, and a trend of more use of hypertonic PD solutions in the App group.

Multimedia Appendix 4 and Table 3 demonstrate differences in the characteristics and treatment of OH among the 3 hospitals with the most clinical interventions for participants at Srinagarind Hospital. The App group significantly received more all modality OH treatment than the No-App group at Srinagarind Hospital. Salt restriction was the principal intervention at all hospitals. Antihypertensive and diuretic medications were commonly administered at Srinagarind Hospital, while hypertonic solutions and adjusted PD prescriptions were frequent treatments at the other 2 hospitals.

**Table 5:** Incidence rate and incidence rate ratio of clinical interventions for overhydration between the App and No-App groups.

Groups				Overall number		Number of cases		Number of events		Incidence rate^a^		IRR^b^		95% CI		*P* value
**All clinical interventions for overhydration**																
		Overall		208		123		637		47.18		—^c^		—		
		App		103		67		473		67.06		1		—		
		No-App		105		56		164		26.74		0.40		0.35-0.46		<.001
**Advice of dietary change**																
		Overall		208		111		578		28.23		—		—		
		App		103		65		436		42.01		1		—		
		No-App		105		46		142		14.06		0.33		0.28-0.40		<.001
**Prescription of hypertensive drugs**																
	Overall		208		44		133		6.50		—		—			
	App		103		28		110		10.60		1		—			
	No-App		105		16		23		2.28		0.21		0.14-0.34		<.001	
**Prescription of diuretic drugs**																
	Overall		208		28		57		2.78		—		—			
	App		103		13		32		3.08		1		—			
	No-App		105		15		25		2.48		0.80		0.48-1.36		.41	
**Prescription of hypertonic solution**																
	Overall		208		68		134		6.54		—		—			
	App		103		34		79		7.61		1		—			
	No-App		105		34		55		5.45		0.72		0.51-1.01		.057	
**Change of PD^d^ prescription**																
	Overall		208		40		60		2.93		—		—			
	App		103		21		35		3.27		1		—			
	No-App		105		19		25		2.48		0.73		0.44-1.23		.24	
**Prescription of 7.5% icodextrin**																
	Overall		208		2		4		0.20		—		—			
	App		103		2		4		0.39		1		—			
	No-App		105		0		0		0.00		N/A^e^		0.00-0.00		.99	

^a^Events per 100 person-month.

^b^IRR: incidence rate ratio.

^c^Not available.

^d^PD: peritoneal dialysis.

^e^N/A: not applicable.

### Secondary Clinical Outcomes

**Figure 2:**
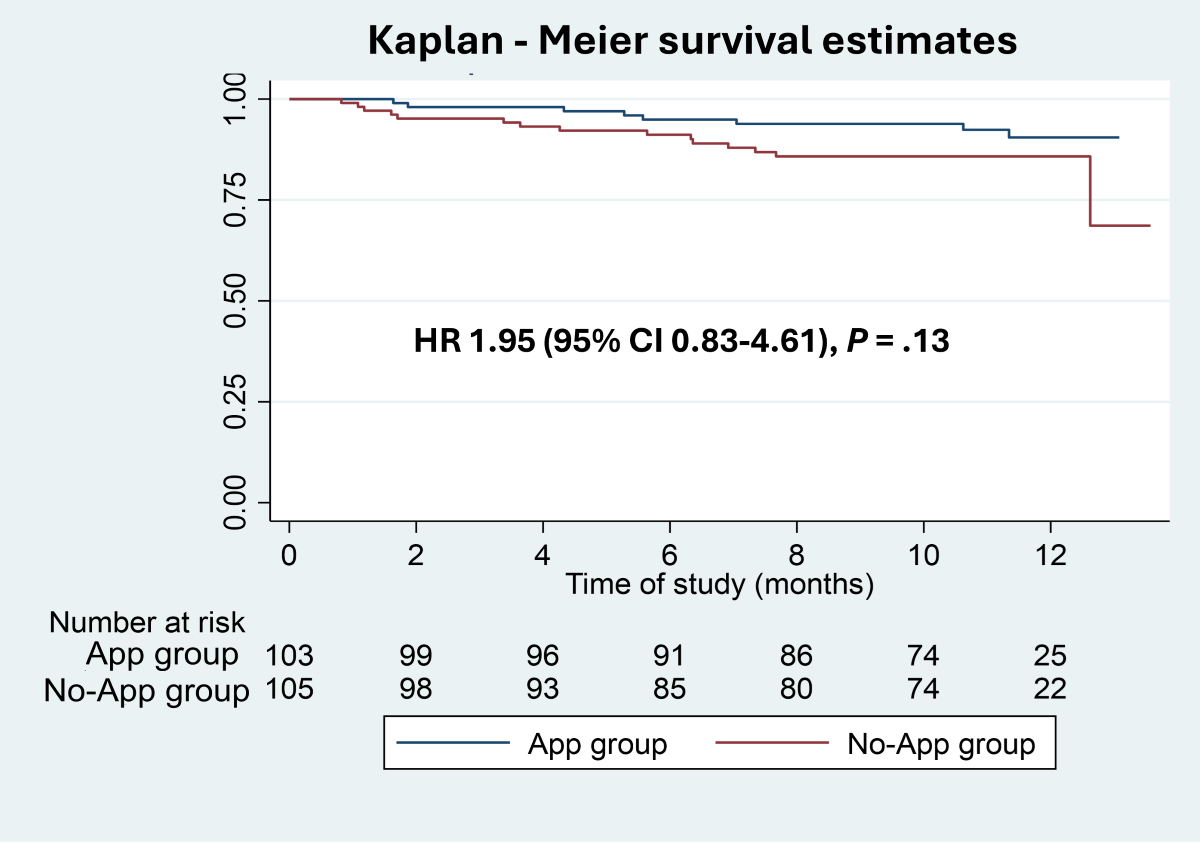
Kaplan-Meier survival analysis between the App and No-App groups. HR: hazard ratio.

The total hospitalization rate ratio for all cause (adjusted IRR 1.60, 95% CI 1.13-2.27), fluid overload (adjusted IRR 4.40, 95% CI 1.26-15.35), and other causes (adjusted IRR 2.31, 95% CI 1.06-5.03) were significantly higher in the No-App group (Table 6). Premature termination in the App group occurred due to death (n=8) and shift to hemodialysis (n=14) compared with the No-App group with death (n=15) and shift to hemodialysis (n=15). The survival estimates between the 2 groups were not statistically significant (hazard ratio 1.95, 95% CI 0.83-4.61; *P*=.13; Figure 2). Laboratory profiles at the last follow-up visits of both groups (App group: n=100 and No-app group: n=98) showed similar results except favorable control of fasting blood sugar in participants with diabetes of the App group (Multimedia Appendix 5).

**Table 6:** Incidence rate per month and incidence rate ratio of hospitalizations.

Groups		Overall number	Number of cases	Number of events	IR^a^	Crude IRR^b^ (95% CI)	*P* value	Adjusted IRR^c^ (95% CI)	*P* value
**Total hospitalization due to any cause**									
	Overall	208	100	180	8.79	—^d^	—	—	—
	App	103	40	67	6.46	1	—	1	—
	No-App	105	60	113	11.19	1.73 (1.28-2.35)	<.001	1.60 (1.13-2.27)	.008
**Hospitalization due to fluid overload**									
	Overall	208	15	21	1.03	—	—	—	—
	App	103	4	5	0.48	1	—	1	—
	No-App	105	11	16	1.58	3.29 (1.21-8.89)	.02	4.40 (1.26-15.35)	.02
**Hospitalization due to peritonitis (PD^e^-related infection)**									
	Overall	208	37	46	2.25	—	—	—	—
	App	103	18	22	2.12	1	—	1	—
	No-App	105	19	24	2.38	1.12 (0.63-2.00)	.70	0.99 (0.50-1.97)	.98
**Hospitalization due to stroke**									
	Overall	208	7	7	0.34	—	—	—	—
	App	103	3	3	0.29	1	—	1	—
	No-App	105	4	4	0.40	1.37 (0.31-6.12)	.68	1.95 (0.32-11.86)	.47
**Hospitalization due to acute coronary syndrome**									
	Overall	208	4	5	0.24	—	—	—	—
	App	103	1	1	0.10	1	—	1	—
	No-App	105	3	4	0.40	4.11 (0.46-36.79)	.21	7.17 (0.24-219.01)	.26
**Hospitalization due to other infections**									
	Overall	208	36	41	2.00	—	—	—	—
	App	103	17	21	2.02	1	—	1	—
	No-App	105	19	20	1.98	0.98 (0.53-1.81)	.95	0.81 (0.41-1.63)	.56
**Hospitalization due to severe anemia**									
	Overall	208	10	13	0.63	—	—	—	—
	App	103	3	3	0.29	1	—	1	—
	No-App	105	7	10	0.99	3.43 (0.94-12.45)	.06	2.96 (0.71-12.29)	.14
**Hospitalization due to other causes**									
	Overall	208	34	47	2.30	—	—	—	—
	App	103	11	12	1.16	1	—	1	—
	No-App	105	23	35	3.47	3.00 (1.56-5.78)	.001	2.31 (1.06-5.03)	.036

^a^IR: incidence rate per 100 person-month.

^b^IRR: incidence rate ratio.

^c^Adjusted with baseline characteristics of age, sex, education levels, hospital levels, underlying diabetes, comorbidities of cardiovascular disease and stroke, systolic and diastolic blood pressure levels, clinical signs of overhydration, hemoglobin values, erythropoietin-stimulating agent use, duration of PD, previous history of peritonitis, exit-site infection, and PD catheter malfunction.

^d^Not applicable.

^e^PD: peritoneal dialysis.

Multivariate analyses revealed important baseline characteristics significantly related to increased hospitalizations including diabetes (for hospitalization from any cause, fluid overload, peritonitis, and other infection), hematocrit (for hospitalization from fluid overload and severe anemia), longer PD vintage (for hospitalization from any cause and peritonitis), and higher DBP (for hospitalization from fluid overload).

## Discussion

### Principal Findings

This is the first RCT in a middle-income country to demonstrate that use of a mobile health app (CKD-PD) can improve detection and treatment of OH in patients on PD resulting in improved BP control, less weight gain, less OH severity, and decrease in hospitalizations from all cause and volume overload. The clinical significance of these findings is supported by research demonstrating that mortality in patients on PD is related to the time of fluid overload [14,18]. Initial fluid overload admission within 12 months, frequent hospitalizations for OH, and a short overall period without fluid overload were significant risk factors of mortality [18]. This suggests the importance of early recognition and aggressive management of OH with effective modalities.

This study was conducted at 3 hospitals located in different socioeconomic locations resulting in disparate baseline participants’ characteristics. Use of the CKD-PD app demonstrated benefit of hospitalization reduction over the No-App group after adjustment with all hospital locations. App users with critical alerts for OH had attenuation in OH severity at Srinagarind and Khon Kaen Hospitals, better control of hypertension at Chaiyaphum and Khon Kaen Hospitals, and reduction of weight gain at Chaiyaphum Hospital. These findings suggest that App users at all 3 sites benefited from more frequent monitoring of hydration status and improved opportunities for early intervention regardless of hospital level and location.

Another important finding from this study is that most OH events in the App group were managed with simpler, lower risk and less expensive treatment interventions than those in the No-App group which had more severe signs and symptoms of OH. The App group OH treatment interventions were mainly salt and water restriction along with antihypertensive medication adjustments compared with the No-App group which required a greater percentage of complex interventions such as diuretics, hypertonic peritoneal solution administration, and change of PD prescription.

A third main finding of this research is that the CKD-PD app facilitated communication between patients on PD and staff with more frequent visits in the App group and use of the integrated telephone call and chat app which improved patients’ self-monitoring and personalized intervention. This supports the use of mobile health intervention to mitigate communication barriers between providers and patients.

### Comparison With Previous Work

Previous studies on the use of telehealth and mobile health apps in patients on PD have focused on high-income countries [19-29]. Commonly used interventions involve remote patient monitoring (RPM), RPM plus video chat, and bidirectional communication between patients and clinic [25]. RPM for patients on PD has been reported in India [21,23] and Japan [30]. However, these studies used home visits, conventional mobile phones, and computers, and did not include real-time visualization of hydration metrics for management of OH.

There have been other studies about the impact of mobile health apps for PD. A recent systematic review of 11 studies from 2013 to 2023 showed that the study designs were diverse [31]. Only 3 of these studies used experimental approaches and the remaining ones used observational-analytic methods and observational-descriptive methods. Most studies reported positive outcomes in patient satisfaction and improving health promotion with these multifunctional mobile health apps. Findings regarding efficiency, effectiveness, impact on mortality, quality of life, and usability were inconsistent across the studies. Some showed significant improvements in laboratory parameters, fluid overload, and solute clearance among app users but no significant differences in self-efficacy, rehospitalization rates, and peritonitis incidence. These outcomes difference may be due to limited numbers of studies and participants, variation in study design, available equipment, targeted outcomes, and inadequate follow-up periods [31].

Diabetes has been identified as the major risk factor of volume overload in patients on PD [18,32,33]. This study supports this as diabetes was a factor related to hospitalizations due to hypervolemia. Increased thirst and water intake in those with diabetes were observed in previous studies, which postulated that hyperglycemia could result in hyperosmolarity, xerostomia, and hyposalivation [33,34], and improvement of OH was noted with salt and water restriction [35]. In addition, diabetes is frequently associated with hypertension, hyperlipidemia, and cardiovascular diseases, which can be related to volume overload and heart failure. This study also found lower hemoglobin levels associated with hospitalization from volume overload. The proposed mechanism may be related to anemia-augmented cardiovascular disease and pulmonary hypertension [36,37].

### Strengths and Limitations

Our study demonstrated multiple strengths: study design (RCT), linkage of RPM with the clinical interventions, and the core outcomes suggested by the SONG-PD (Standardized Outcomes in Nephrology-Peritoneal Dialysis) consensus. We also included different hospital types and populations.

There are 3 limitations to this study. First, the number of patients on PD at each of the hospital sites was not enough to stratify and compare patients from different settings for all outcomes. Second, the median follow-up duration was 11.2 months with 70% (73/105) of participants of the No-App group and 75% (77/103) of participants of the App group completing the entire study; therefore, premature termination might affect our results. Third, although reduction of overall and OH hospitalizations was observed in this study, no significant decrease in mortality was demonstrated, likely due to inadequate sample size.

### Future Directions

A future larger-scale efficiency RCT is the next step to evaluating the CKD-PD app. This will address sample size and early termination limitations and allow for a more rigorous evaluation of our intervention with more participants at different types of study sites. It would also provide the opportunity to develop a robust PD database that could be used to develop predictive algorithms for when and how to optimize monitoring and clinical interventions for OH. Additional research into cost-effectiveness is another important study for direct resources and policy regarding the use of mobile health technology and RPM for patients on PD.

### Conclusions

Use of the CKD-PD app can improve early detection OH in patients on PD using near real-time monitoring and communication with the treatment team. Patients can be treated with simpler, less expensive treatments and have fewer all-cause and volume overload hospitalizations in a middle-income country where PD is widely used.

## Appendix

Multimedia Appendix 1 CONSORT-eHEALTH checklist (V 1.6.1).

Multimedia Appendix 2 Chronic Kidney Disease—Peritoneal Dialysis app screenshots.

Multimedia Appendix 3 Baseline characteristics of participants at the 3 hospitals.

Multimedia Appendix 4 Incidence rate ratio of interventions for overhydration between the App and No-App groups at the 3 hospitals.

Multimedia Appendix 5 Laboratory profiles at the last hospital visits of participants.

## Abbreviations

BP: blood pressure
BW: body weight
CKD: chronic kidney disease
CKDNET: Chronic Kidney Disease Prevention in the Northeast of Thailand
CONSORT-EHEALTH: Consolidated Standards of Reporting Trials of Electronic and Mobile Health Applications and Online Telehealth
IR: incidence rate
IRR: incidence rate ratio
KF: kidney failure
OH: overhydration
PD: peritoneal dialysis
RCT: randomized controlled trial
RPM: remote patient monitoring
SONG-PD: the Standardized Outcomes in Nephrology-Peritoneal Dialysis
UF: ultrafiltration

## Data Availability

All extracted and analyzed data are available on reasonable request to the corresponding author.
