# Supplementary material for: Effect of the Chronic Kidney Disease—Peritoneal Dialysis (CKD-PD) App on Improvement of Overhydration Treatment in Patients on Peritoneal Dialysis: Randomized Controlled Trial
Source: J Med Internet Res. 2025 May 21;27:e70641. doi: 10.2196/70641 (PMC12138318; doi:10.2196/70641)
Supplement: Multimedia Appendix 1 [file jmir_v27i1e70641_app1.pdf]

CONSORT-EHEALTH (V 1.6.1) - Submission/Publication Form

The CONSORT-EHEALTH checklist is intended for authors of randomized trials evaluating web-based and Internet-based applications/interventions, including mobile interventions, electronic games (incl multiplayer games), social media, certain telehealth applications, and other interactive and/or networked electronic applications. Some of the items (e.g. all subitems under item 5 - description of the intervention) may also be applicable for other study designs.

The goal of the CONSORT EHEALTH checklist and guideline is to be
a) a guide for reporting for authors of RCTs,
b) to form a basis for appraisal of an ehealth trial (in terms of validity)

CONSORT-EHEALTH items/subitems are MANDATORY reporting items for studies published in the Journal of Medical Internet Research and other journals / scientific societies endorsing the checklist.

Items numbered 1., 2., 3., 4a., 4b etc are original CONSORT or CONSORT-NPT (non-pharmacologic treatment) items.
Items with Roman numerals (i., ii, iii, iv etc.) are CONSORT-EHEALTH extensions/clarifications.

As the CONSORT-EHEALTH checklist is still considered in a formative stage, we would ask that you also RATE ON A SCALE OF 1-5 how important/useful you feel each item is FOR THE PURPOSE OF THE CHECKLIST and reporting guideline (optional).

Mandatory reporting items are marked with a red *.
In the textboxes, either copy & paste the relevant sections from your manuscript into this form - please include any quotes from your manuscript in QUOTATION MARKS,
or answer directly by providing additional information not in the manuscript, or elaborating on why the item was not relevant for this study.

YOUR ANSWERS WILL BE PUBLISHED AS A SUPPLEMENTARY FILE TO YOUR PUBLICATION IN JMIR AND ARE CONSIDERED PART OF YOUR PUBLICATION (IF ACCEPTED).
Please fill in these questions diligently. Information will not be copyedited, so please use proper spelling and grammar, use correct capitalization, and avoid abbreviations.

DO NOT FORGET TO SAVE AS PDF _AND_ CLICK THE SUBMIT BUTTON SO YOUR ANSWERS ARE IN OUR DATABASE !!!

Citation Suggestion (if you append the pdf as Appendix we suggest to cite this paper in the caption):
Eysenbach G, CONSORT-EHEALTH Group
CONSORT-EHEALTH: Improving and Standardizing Evaluation Reports of Web-based and Mobile Health Interventions
J Med Internet Res 2011;13(4):e126

URL: http://www.jmir.org/2011/4/e126/
doi: 10.2196/jmir.1923
PMID: 22209829

sirirt_a@kku.ac.th Switch account

Not shared

Draft saved

* Indicates required question

Your name *

First Last

Sirirat Anutrakulchai

Primary Affiliation (short), City, Country *

University of Toronto, Toronto, Canada

Khon Kaen University, Khon Kaen, Thailand

Your e-mail address *

abc@gmail.com

sirirt_a@kku.ac.th

Title of your manuscript *

Provide the (draft) title of your manuscript.

Effect of CKD-PD App on Improvement of Overhydration Treatment in Patients on Peritoneal Dialysis: A Randomized Controlled Trial

Name of your App/Software/Intervention *

If there is a short and a long/alternate name, write the short name first and add the long name in brackets.

CKD-PD App (Chronic Kidney Disease – Peritor

Evaluated Version (if any)

e.g. "V1", "Release 2017-03-01", "Version 2.0.27913"

Version 15.1.14

Language(s) *

What language is the intervention/app in? If multiple languages are available, separate by comma (e.g. "English, French")

Thai language

URL of your Intervention Website or App

e.g. a direct link to the mobile app on app in appstore (itunes, Google Play), or URL of the website. If the intervention is a DVD or hardware, you can also link to an Amazon page.

https://apps.apple.com/us/app/ckd-%E0%B8%A3-%E0%B8%81%E0%B8%A9-%E0%B9%84%E0

URL of an image/screenshot (optional)

https://apps.apple.com/us/app/ckd-%E0%B8%A3-%E0%B8%81%E0%B8%A9-%E0%B9%84%E0

Accessibility *

Can an enduser access the intervention presently?

- (●) access is free and open
- ( ) access only for special usergroups, not open
- ( ) access is open to everyone, but requires payment/subscription/in-app purchases
- ( ) app/intervention no longer accessible
- ( ) Other:

Primary Medical Indication/Disease/Condition *

e.g. "Stress", "Diabetes", or define the target group in brackets after the condition, e.g. "Autism (Parents of children with)", "Alzheimers (Informal Caregivers of)"

"Volume overload in End Stage Kidney Disease

Primary Outcomes measured in trial *

comma-separated list of primary outcomes reported in the trial

The incidence rate ratio (IRR) for clinical interv

Secondary/other outcomes

Are there any other outcomes the intervention is expected to affect?

Hospitalizations, technique failure, and death

Recommended "Dose" *

What do the instructions for users say on how often the app should be used?

- (x) Approximately Daily
- ( ) Approximately Weekly
- ( ) Approximately Monthly
- ( ) Approximately Yearly
- ( ) "as needed"
- ( ) Other:

Approx. Percentage of Users (starters) still using the app as recommended after 3 months *

- ( ) unknown / not evaluated
- ( ) 0-10%
- ( ) 11-20%
- ( ) 21-30%
- ( ) 31-40%
- ( ) 41-50%
- ( ) 51-60%
- ( ) 61-70%
- ( ) 71%-80%
- (x) 81-90%
- ( ) 91-100%
- ( ) Other:

Overall, was the app/intervention effective? *

- [x] yes: all primary outcomes were significantly better in intervention group vs control
- [ ] partly: SOME primary outcomes were significantly better in intervention group vs control
- [ ] no statistically significant difference between control and intervention
- [ ] potentially harmful: control was significantly better than intervention in one or more outcomes
- [ ] inconclusive: more research is needed
- [ ] Other:

Article Preparation Status/Stage *

At which stage in your article preparation are you currently (at the time you fill in this form)

- [ ] not submitted yet - in early draft status
- [x] not submitted yet - in late draft status, just before submission
- [ ] submitted to a journal but not reviewed yet
- [ ] submitted to a journal and after receiving initial reviewer comments
- [ ] submitted to a journal and accepted, but not published yet
- [ ] published
- [ ] Other:

Journal *

If you already know where you will submit this paper (or if it is already submitted), please provide the journal name (if it is not JMIR, provide the journal name under "other")

- ○ not submitted yet / unclear where I will submit this
- ◉ Journal of Medical Internet Research (JMIR)
- ○ JMIR mHealth and UHealth
- ○ JMIR Serious Games
- ○ JMIR Mental Health
- ○ JMIR Public Health
- ○ JMIR Formative Research
- ○ Other JMIR sister journal
- ○ Other:

Is this a full powered effectiveness trial or a pilot/feasibility trial? *

- ○ Pilot/feasibility
- ◉ Fully powered

Manuscript tracking number *

If this is a JMIR submission, please provide the manuscript tracking number under "other" (The ms tracking number can be found in the submission acknowledgement email, or when you login as author in JMIR. If the paper is already published in JMIR, then the ms tracking number is the four-digit number at the end of the DOI, to be found at the bottom of each published article in JMIR)

- ◉ no ms number (yet) / not (yet) submitted to / published in JMIR
- ○ Other:

TITLE AND ABSTRACT

1a) TITLE: Identification as a randomized trial in the title

1a) Does your paper address CONSORT item 1a? *

I.e does the title contain the phrase "Randomized Controlled Trial"? (if not, explain the reason under "other")

- (selected) yes
- ( ) Other:

1a-i) Identify the mode of delivery in the title

Identify the mode of delivery. Preferably use “web-based” and/or “mobile” and/or “electronic game” in the title. Avoid ambiguous terms like “online”, “virtual”, “interactive”. Use “Internet-based” only if Intervention includes non-web-based Internet components (e.g. email), use “computer-based” or “electronic” only if offline products are used. Use “virtual” only in the context of “virtual reality” (3-D worlds). Use “online” only in the context of “online support groups”. Complement or substitute product names with broader terms for the class of products (such as “mobile” or “smart phone” instead of “iphone”), especially if the application runs on different platforms.

	1	2	3	4	5	
subitem not at all important	○	○	○	○	●	essential

Clear selection

Does your paper address subitem 1a-i? *

Copy and paste relevant sections from manuscript title (include quotes in quotation marks "like this" to indicate direct quotes from your manuscript), or elaborate on this item by providing additional information not in the ms, or briefly explain why the item is not applicable/relevant for your study

"Effect of CKD-PD App on Improvement of Overhydration Treatment in Patients on Peritoneal Dialysis: A Randomized Controlled Trial"

1a-ii) Non-web-based components or important co-interventions in title

Mention non-web-based components or important co-interventions in title, if any (e.g., “with telephone support”).

	1	2	3	4	5	
subitem not at all important	○	○	●	○	○	essential

Clear selection

Does your paper address subitem 1a-ii?

Copy and paste relevant sections from manuscript title (include quotes in quotation marks "like this" to indicate direct quotes from your manuscript), or elaborate on this item by providing additional information not in the ms, or briefly explain why the item is not applicable/relevant for your study

"Effect of CKD-PD App on Improvement of Overhydration Treatment in Patients on Peritoneal Dialysis: A Randomized Controlled Trial"

1a-iii) Primary condition or target group in the title

Mention primary condition or target group in the title, if any (e.g., “for children with Type I Diabetes”) Example: A Web-based and Mobile Intervention with Telephone Support for Children with Type I Diabetes: Randomized Controlled Trial

	1	2	3	4	5	
subitem not at all important	○	○	○	○	●	essential

Clear selection

Does your paper address subitem 1a-iii? *

Copy and paste relevant sections from manuscript title (include quotes in quotation marks "like this" to indicate direct quotes from your manuscript), or elaborate on this item by providing additional information not in the ms, or briefly explain why the item is not applicable/relevant for your study

"Overhydration Treatment in Patients on Peritoneal Dialysis"

1b) ABSTRACT: Structured summary of trial design, methods, results, and conclusions

NPT extension: Description of experimental treatment, comparator, care providers, centers, and blinding status.

1b-i) Key features/functionalities/components of the intervention and comparator in the METHODS section of the ABSTRACT

Mention key features/functionalities/components of the intervention and comparator in the abstract. If possible, also mention theories and principles used for designing the site. Keep in mind the needs of systematic reviewers and indexers by including important synonyms. (Note: Only report in the abstract what the main paper is reporting. If this information is missing from the main body of text, consider adding it)

	1	2	3	4	5	
subitem not at all important	○	○	○	○	◉	essential

Clear selection

Does your paper address subitem 1b-i? *

Copy and paste relevant sections from the manuscript abstract (include quotes in quotation marks "like this" to indicate direct quotes from your manuscript), or elaborate on this item by providing additional information not in the ms, or briefly explain why the item is not applicable/relevant for your study

"An open-label randomized control trial was conducted at three hospitals in northeast Thailand. Enrolled participants from PD clinics were randomized into two equal groups: CKD-PD App users, and usual management (No-App)."

1b-ii) Level of human involvement in the METHODS section of the ABSTRACT

Clarify the level of human involvement in the abstract, e.g., use phrases like “fully automated” vs. “therapist/nurse/care provider/physician-assisted” (mention number and expertise of providers involved, if any). (Note: Only report in the abstract what the main paper is reporting. If this information is missing from the main body of text, consider adding it)

	1	2	3	4	5	
subitem not at all important	○	○	○	○	◉	essential

Clear selection

Does your paper address subitem 1b-ii?

Copy and paste relevant sections from the manuscript abstract (include quotes in quotation marks "like this" to indicate direct quotes from your manuscript), or elaborate on this item by providing additional information not in the ms, or briefly explain why the item is not applicable/relevant for your study

"Participants or their caregivers in the App group recorded hydration metrics in the CKD-PD app, uploaded to a central database monitored by nephrology staff. The No-App group used a handwritten logbook."

1b-iii) Open vs. closed, web-based (self-assessment) vs. face-to-face assessments in the METHODS section of the ABSTRACT

Mention how participants were recruited (online vs. offline), e.g., from an open access website or from a clinic or a closed online user group (closed usergroup trial), and clarify if this was a purely web-based trial, or there were face-to-face components (as part of the intervention or for assessment). Clearly say if outcomes were self-assessed through questionnaires (as common in web-based trials). Note: In traditional offline trials, an open trial (open-label trial) is a type of clinical trial in which both the researchers and participants know which treatment is being administered. To avoid confusion, use “blinded” or “unblinded” to indicated the level of blinding instead of “open”, as “open” in web-based trials usually refers to “open access” (i.e. participants can self-enrol). (Note: Only report in the abstract what the main paper is reporting. If this information is missing from the main body of text, consider adding it)

	1	2	3	4	5	
subitem not at all important	○	○	○	○	◉	essential

Clear selection

Does your paper address subitem 1b-iii?

Copy and paste relevant sections from the manuscript abstract (include quotes in quotation marks "like this" to indicate direct quotes from your manuscript), or elaborate on this item by providing additional information not in the ms, or briefly explain why the item is not applicable/relevant for your study

"An open-label randomized control trial was conducted at three hospitals in northeast Thailand. Enrolled participants from PD clinics were randomized into two equal groups: CKD-PD App users, and usual management (No-App). Participants or their caregivers in the App group recorded hydration metrics in the CKD-PD app, uploaded to a central database monitored by nephrology staff. The No-App group used a handwritten logbook. Both groups had bimonthly clinic visits."

1b-iv) RESULTS section in abstract must contain use data

Report number of participants enrolled/assessed in each group, the use/uptake of the intervention (e.g., attrition/adherence metrics, use over time, number of logins etc.), in addition to primary/secondary outcomes. (Note: Only report in the abstract what the main paper is reporting. If this information is missing from the main body of text, consider adding it)

	1	2	3	4	5	
subitem not at all important	○	○	○	○	◉	essential

Clear selection

Does your paper address subitem 1b-iv?

Copy and paste relevant sections from the manuscript abstract (include quotes in quotation marks "like this" to indicate direct quotes from your manuscript), or elaborate on this item by providing additional information not in the ms, or briefly explain why the item is not applicable/relevant for your study

"208 participants were randomized into App (N=103) and No-App (N=105) groups with the median followed-up time 11.2 months. Hydration metric upload compliance in the App group was 85.7 % (71.4 - 95.6)."

1b-v) CONCLUSIONS/DISCUSSION in abstract for negative trials

Conclusions/Discussions in abstract for negative trials: Discuss the primary outcome - if the trial is negative (primary outcome not changed), and the intervention was not used, discuss whether negative results are attributable to lack of uptake and discuss reasons. (Note: Only report in the abstract what the main paper is reporting. If this information is missing from the main body of text, consider adding it)

	1	2	3	4	5	
subitem not at all important	○	○	○	○	◉	essential

Clear selection

Does your paper address subitem 1b-v?

Copy and paste relevant sections from the manuscript abstract (include quotes in quotation marks "like this" to indicate direct quotes from your manuscript), or elaborate on this item by providing additional information not in the ms, or briefly explain why the item is not applicable/relevant for your study

"Use of the CKD-PD app improved hydration status monitoring. Contact with personal led to early detection and management of overhydration, and a decrease in all cause and volume overload hospitalizations."

INTRODUCTION

2a) In INTRODUCTION: Scientific background and explanation of rationale

2a-i) Problem and the type of system/solution

Describe the problem and the type of system/solution that is object of the study: intended as stand-alone intervention vs. incorporated in broader health care program? Intended for a particular patient population? Goals of the intervention, e.g., being more cost-effective to other interventions, replace or complement other solutions? (Note: Details about the intervention are provided in “Methods” under 5)

	1	2	3	4	5	
subitem not at all important	○	○	○	○	◉	essential

Clear selection

Does your paper address subitem 2a-i? *

Copy and paste relevant sections from the manuscript (include quotes in quotation marks "like this" to indicate direct quotes from your manuscript), or elaborate on this item by providing additional information not in the ms, or briefly explain why the item is not applicable/relevant for your study

"Overhydration (OH) is one of the most common complications in PD patients. Clinical manifestations of OH include peripheral edema, dyspnea, and hypertension. It is estimated that 50-60% of PD patients are overhydrated, with severe OH producing clinical symptoms in 25%. OH is associated with increased morbidity and mortality due to infections including peritonitis, and major adverse cardiovascular events such as myocardial infarction, pulmonary edema, stroke, and hypertensive crisis. OH alone, independent of coexisting cardiovascular pathologies, is a risk factor for increased morbidity and mortality. Early detection of OH is possible by monitoring patients' hydration metrics."

2a-ii) Scientific background, rationale: What is known about the (type of) system

Scientific background, rationale: What is known about the (type of) system that is the object of the study (be sure to discuss the use of similar systems for other conditions/diagnoses, if appropiate), motivation for the study, i.e. what are the reasons for and what is the context for this specific study, from which stakeholder viewpoint is the study performed, potential impact of findings [2]. Briefly justify the choice of the comparator.

	1	2	3	4	5	
subitem not at all important	○	○	○	○	◉	essential

Clear selection

Does your paper address subitem 2a-ii? *

Copy and paste relevant sections from the manuscript (include quotes in quotation marks "like this" to indicate direct quotes from your manuscript), or elaborate on this item by providing additional information not in the ms, or briefly explain why the item is not applicable/relevant for your study

"Early detection of OH is possible by monitoring patients' hydration metrics. In northeast Thailand, nephrologists and PD nurses routinely assess PD patients' hydration status by reviewing hydration metrics – blood pressure (BP), body weight (BW), and ultrafiltration volume (UF) collected and recorded by patients in a handwritten logbook during scheduled bimonthly clinic visits. The critical gap for the treatment of OH lies in obtaining these hydration metrics in a timely and actionable format when early interventions can be made to improve quality of life, decrease health care costs, and reduce morbidity and mortality."

2b) In INTRODUCTION: Specific objectives or hypotheses

Does your paper address CONSORT subitem 2b? *

Copy and paste relevant sections from the manuscript (include quotes in quotation marks "like this" to indicate direct quotes from your manuscript), or elaborate on this item by providing additional information not in the ms, or briefly explain why the item is not applicable/relevant for your study

"The objective of this study was to compare the detection of OH and clinical outcomes in PD patients using the CKD-PD app with the standard monitoring and management of PD patients."

METHODS

3a) Description of trial design (such as parallel, factorial) including allocation ratio

Does your paper address CONSORT subitem 3a? *

Copy and paste relevant sections from the manuscript (include quotes in quotation marks "like this" to indicate direct quotes from your manuscript), or elaborate on this item by providing additional information not in the ms, or briefly explain why the item is not applicable/relevant for your study

"This study was an open-label randomized control trial conducted between December 2021 and February 2023 at the three hospitals in northeast Thailand: 1) Srinagarind Hospital (academic tertiary) 2) Khon Kaen Hospital (urban provincial), and 3) Chaiyaphum Hospital (rural provincial). ESKD patients on PD at clinics of these hospitals were enrolled using the inclusion criteria: willingness to participate, age ≥ 18 years, access to a smartphone, and the ability to use the CKD-PD app (independently or with a surrogate). Randomization codes for the two groups were generated using computer software. Block randomization with varying block sizes of 2 and 4 was employed to ensure allocation concealment and balance between the arms. The randomization sequence was created using a random number generator and was implemented without stratification. The allocation sequence was concealed from study personnel and participants until assignment. All enrolled participants provided written informed consent and were randomized into two equal groups at each hospital, one using the CKD-PD app ("App" group), and one receiving usual management ("No-App" group)."

3b) Important changes to methods after trial commencement (such as eligibility criteria), with reasons

Does your paper address CONSORT subitem 3b? *

Copy and paste relevant sections from the manuscript (include quotes in quotation marks "like this" to indicate direct quotes from your manuscript), or elaborate on this item by providing additional information not in the ms, or briefly explain why the item is not applicable/relevant for your study

No change of the methods occurred

3b-i) Bug fixes, Downtimes, Content Changes

Bug fixes, Downtimes, Content Changes: ehealth systems are often dynamic systems. A description of changes to methods therefore also includes important changes made on the intervention or comparator during the trial (e.g., major bug fixes or changes in the functionality or content) (5-iii) and other “unexpected events” that may have influenced study design such as staff changes, system failures/downtimes, etc. [2].

	1	2	3	4	5	
subitem not at all important	○	○	◉	○	○	essential

Clear selection

Does your paper address subitem 3b-i?

Copy and paste relevant sections from the manuscript (include quotes in quotation marks "like this" to indicate direct quotes from your manuscript), or elaborate on this item by providing additional information not in the ms, or briefly explain why the item is not applicable/relevant for your study

"Participants were provided internet access in cases of their internet signal instabilities and hydration metrics were temporarily sent via LINE® program if unexpected problems of CKD-PD app happened such as infrequent downtimes."

4a) Eligibility criteria for participants

Does your paper address CONSORT subitem 4a? *

Copy and paste relevant sections from the manuscript (include quotes in quotation marks "like this" to indicate direct quotes from your manuscript), or elaborate on this item by providing additional information not in the ms, or briefly explain why the item is not applicable/relevant for your study

"ESKD patients on PD at clinics of these hospitals were enrolled using the inclusion criteria: willingness to participate, age ≥ 18 years, access to a smartphone, and the ability to use the CKD-PD app (independently or with a surrogate)."

4a-i) Computer / Internet literacy

Computer / Internet literacy is often an implicit “de facto” eligibility criterion - this should be explicitly clarified.

	1	2	3	4	5	
subitem not at all important	○	○	○	○	◉	essential

Clear selection

Does your paper address subitem 4a-i?

Copy and paste relevant sections from the manuscript (include quotes in quotation marks "like this" to indicate direct quotes from your manuscript), or elaborate on this item by providing additional information not in the ms, or briefly explain why the item is not applicable/relevant for your study

"The inclusion criteria: willingness to participate, age ≥ 18 years, access to a smartphone, and the ability to use the CKD-PD app (independently or with a surrogate)."

4a-ii) Open vs. closed, web-based vs. face-to-face assessments:

Open vs. closed, web-based vs. face-to-face assessments: Mention how participants were recruited (online vs. offline), e.g., from an open access website or from a clinic, and clarify if this was a purely web-based trial, or there were face-to-face components (as part of the intervention or for assessment), i.e., to what degree got the study team to know the participant. In online-only trials, clarify if participants were quasi-anonymous and whether having multiple identities was possible or whether technical or logistical measures (e.g., cookies, email confirmation, phone calls) were used to detect/prevent these.

	1	2	3	4	5	
subitem not at all important	○	○	○	○	◉	essential

Clear selection

Does your paper address subitem 4a-ii? *

Copy and paste relevant sections from the manuscript (include quotes in quotation marks "like this" to indicate direct quotes from your manuscript), or elaborate on this item by providing additional information not in the ms, or briefly explain why the item is not applicable/relevant for your study

"ESKD patients on PD at clinics of these hospitals were enrolled"
"All participants continued standard bimonthly PD clinic visits during the study period."
"Participants were assigned a PD clinic visit appointment date based on their randomization allocation to reduce contamination between the two study groups."
"Participants were instructed to record their hydration metrics data daily. The PD clinic staff checked participant hydration metric data in the CKDNET database weekly. If the hydration metrics were not uploaded, the PD staff reminded the participant by telephone or chat application."

4a-iii) Information giving during recruitment

Information given during recruitment. Specify how participants were briefed for recruitment and in the informed consent procedures (e.g., publish the informed consent documentation as appendix, see also item X26), as this information may have an effect on user self-selection, user expectation and may also bias results.

	1	2	3	4	5	
subitem not at all important	○	○	○	○	◉	essential

Clear selection

Does your paper address subitem 4a-iii?

Copy and paste relevant sections from the manuscript (include quotes in quotation marks "like this" to indicate direct quotes from your manuscript), or elaborate on this item by providing additional information not in the ms, or briefly explain why the item is not applicable/relevant for your study

"All enrolled participants provided written informed consent and were randomized into two equal groups at each hospital, one using the CKD-PD app (“App” group), and one receiving usual management (“No-App” group)."

4b) Settings and locations where the data were collected

Does your paper address CONSORT subitem 4b? *

Copy and paste relevant sections from the manuscript (include quotes in quotation marks "like this" to indicate direct quotes from your manuscript), or elaborate on this item by providing additional information not in the ms, or briefly explain why the item is not applicable/relevant for your study

"Hydration metric collection in both groups was performed by participants or their surrogate daily and included 1) morning BP 2) BW before first dialysis cycle 3) UF volume: difference in total weight of the dialysate fluid bags before and after each peritoneal dwell period for the preceding 24 hours. The No-App group received usual care: 1) “PD logbook”- a handwritten logbook to record hydration metrics, 2) bimonthly PD clinic appointments and 3) instructions to contact the clinic for any concerns. No other outreach was conducted unless the participant contacted the PD staff or sought care at the PD clinic or emergency department."
"The App group received training on how to use the CKD-PD app including hydration metric entry, self-monitoring, and in-app communication features. Participants were instructed to record their hydration metrics data daily. The PD clinic staff checked participant hydration metric data in the CKDNET database weekly. If the hydration metrics were not uploaded, the PD staff reminded the participant by telephone or chat application. "

4b-i) Report if outcomes were (self-)assessed through online questionnaires

Clearly report if outcomes were (self-)assessed through online questionnaires (as common in web-based trials) or otherwise.

	1	2	3	4	5	
subitem not at all important	○	○	○	○	◉	essential

Clear selection

Does your paper address subitem 4b-i? *

Copy and paste relevant sections from the manuscript (include quotes in quotation marks "like this" to indicate direct quotes from your manuscript), or elaborate on this item by providing additional information not in the ms, or briefly explain why the item is not applicable/relevant for your study

"The App group received training on how to use the CKD-PD app including hydration metric entry, self-monitoring, and in-app communication features. Participants were instructed to record their hydration metrics data daily. The PD clinic staff checked participant hydration metric data in the CKDNET database weekly. If the hydration metrics were not uploaded, the PD staff reminded the participant by telephone or chat application."

4b-ii) Report how institutional affiliations are displayed

Report how institutional affiliations are displayed to potential participants [on ehealth media], as affiliations with prestigious hospitals or universities may affect volunteer rates, use, and reactions with regards to an intervention.(Not a required item – describe only if this may bias results)

	1	2	3	4	5	
subitem not at all important	◉	○	○	○	○	essential

Clear selection

Does your paper address subitem 4b-ii?

Copy and paste relevant sections from the manuscript (include quotes in quotation marks "like this" to indicate direct quotes from your manuscript), or elaborate on this item by providing additional information not in the ms, or briefly explain why the item is not applicable/relevant for your study

All three sites had similar volunteer rates.

5) The interventions for each group with sufficient details to allow replication, including how and when they were actually administered

5-i) Mention names, credential, affiliations of the developers, sponsors, and owners

Mention names, credential, affiliations of the developers, sponsors, and owners [6] (if authors/evaluators are owners or developer of the software, this needs to be declared in a "Conflict of interest" section or mentioned elsewhere in the manuscript).

	1	2	3	4	5	
subitem not at all important	○	○	○	○	◉	essential

Clear selection

Does your paper address subitem 5-i?

Copy and paste relevant sections from the manuscript (include quotes in quotation marks "like this" to indicate direct quotes from your manuscript), or elaborate on this item by providing additional information not in the ms, or briefly explain why the item is not applicable/relevant for your study

"Bandit Thinkhamrop is one of the developers of CKD-PD app, however, he was not involved in the process of methodology such as data curation, investigation, collection, and statistical analysis."

5-ii) Describe the history/development process

Describe the history/development process of the application and previous formative evaluations (e.g., focus groups, usability testing), as these will have an impact on adoption/use rates and help with interpreting results.

	1	2	3	4	5	
subitem not at all important	○	○	○	○	◉	essential

Clear selection

Does your paper address subitem 5-ii?

Copy and paste relevant sections from the manuscript (include quotes in quotation marks "like this" to indicate direct quotes from your manuscript), or elaborate on this item by providing additional information not in the ms, or briefly explain why the item is not applicable/relevant for your study

"Hydration metrics including BP, BW, UF volume, were recorded in the CKD-PD app and uploaded to the Chronic Kidney Disease Prevention in the Northeast of Thailand (CKDNET) database in the Thai Care Cloud (TCC).The data are graphically displayed on both the CKD-PD app and TCC website and accessible by PD clinic staff. The key features of the CKD-PD app are: 1) daily data entry for continuous monitoring of hydration metrics, 2) graphical display of hydration status for app users, 3) near real-time data and alerts for clinic staff about critical abnormalities in patients' hydration metrics, 4) an integrated secure chat function (LINE® professional) to facilitate communication between PD patients and clinic staff, 5) integration of hydration metrics from the CKD-PD app with electronic health data in the CKDNET database on the TCC. The CKD-PD app was assessed by focus groups and usability testing processed."

5-iii) Revisions and updating

Revisions and updating. Clearly mention the date and/or version number of the application/intervention (and comparator, if applicable) evaluated, or describe whether the intervention underwent major changes during the evaluation process, or whether the development and/or content was "frozen" during the trial. Describe dynamic components such as news feeds or changing content which may have an impact on the replicability of the intervention (for unexpected events see item 3b).

	1	2	3	4	5	
subitem not at all important	○	○	◉	○	○	essential

Clear selection

Does your paper address subitem 5-iii?

Copy and paste relevant sections from the manuscript (include quotes in quotation marks "like this" to indicate direct quotes from your manuscript), or elaborate on this item by providing additional information not in the ms, or briefly explain why the item is not applicable/relevant for your study

No change of app version during the study period

5-iv) Quality assurance methods

Provide information on quality assurance methods to ensure accuracy and quality of information provided [1], if applicable.

	1	2	3	4	5	
subitem not at all important	○	○	●	○	○	essential

Clear selection

Does your paper address subitem 5-iv?

Copy and paste relevant sections from the manuscript (include quotes in quotation marks "like this" to indicate direct quotes from your manuscript), or elaborate on this item by providing additional information not in the ms, or briefly explain why the item is not applicable/relevant for your study

"The App group received training on how to use the CKD-PD app including hydration metric entry, self-monitoring, and in-app communication features. Participants were instructed to record their hydration metrics data daily. The PD clinic staff checked participant hydration metric data in the CKDNET database weekly. If the hydration metrics were not uploaded, the PD staff reminded the participant by telephone or chat application."

5-v) Ensure replicability by publishing the source code, and/or providing screenshots/screen-capture video, and/or providing flowcharts of the algorithms used

Ensure replicability by publishing the source code, and/or providing screenshots/screen-capture video, and/or providing flowcharts of the algorithms used. Replicability (i.e., other researchers should in principle be able to replicate the study) is a hallmark of scientific reporting.

	1	2	3	4	5	
subitem not at all important	○	○	○	○	●	essential

Clear selection

Does your paper address subitem 5-v?

Copy and paste relevant sections from the manuscript (include quotes in quotation marks "like this" to indicate direct quotes from your manuscript), or elaborate on this item by providing additional information not in the ms, or briefly explain why the item is not applicable/relevant for your study

"Multimedia Appendix 1: CKD-PD app Screenshots"

5-vi) Digital preservation

Digital preservation: Provide the URL of the application, but as the intervention is likely to change or disappear over the course of the years; also make sure the intervention is archived (Internet Archive, webcitation.org, and/or publishing the source code or screenshots/videos alongside the article). As pages behind login screens cannot be archived, consider creating demo pages which are accessible without login.

	1	2	3	4	5	
subitem not at all important	○	○	○	○	●	essential

Clear selection

Does your paper address subitem 5-vi?

Copy and paste relevant sections from the manuscript (include quotes in quotation marks "like this" to indicate direct quotes from your manuscript), or elaborate on this item by providing additional information not in the ms, or briefly explain why the item is not applicable/relevant for your study

"https://apps.apple.com/us/app/ckd-%E0%B8%A3-%E0%B8%81%E0%B8%A9-%E0%B9%84%E0%B8%95/id1332960784"
"Multimedia Appendix 1: CKD-PD app Screenshots"

5-vii) Access

Access: Describe how participants accessed the application, in what setting/context, if they had to pay (or were paid) or not, whether they had to be a member of specific group. If known, describe how participants obtained “access to the platform and Internet” [1]. To ensure access for editors/reviewers/readers, consider to provide a “backdoor” login account or demo mode for reviewers/readers to explore the application (also important for archiving purposes, see vi).

	1	2	3	4	5	
subitem not at all important	○	○	○	○	◉	essential

Clear selection

Does your paper address subitem 5-vii? *

Copy and paste relevant sections from the manuscript (include quotes in quotation marks "like this" to indicate direct quotes from your manuscript), or elaborate on this item by providing additional information not in the ms, or briefly explain why the item is not applicable/relevant for your study

"The CKD-PD app is available for free download in both Android and iOS formats. It is developed for individuals who want to track and monitor their own renal status, BP, BW, blood sugar levels, water intake and output, and the PD status (if relevance). In case of subjects allowed to send their data recorded at home to the cloud system and then seen and monitored by the involved personnel, they will receive username and password for getting into the app system."

"In this study, hydration metrics including BP, BW, UF volume, were recorded in the CKD-PD app and uploaded to the Chronic Kidney Disease Prevention in the Northeast of Thailand (CKDNET) database in the Thai Care Cloud (TCC).16,17 The data are graphically displayed on both the CKD-PD app and TCC website and accessible by PD clinic staff. The key features of the CKD-PD app are: 1) daily data entry for continuous monitoring of hydration metrics, 2) graphical display of hydration status for app users, 3) near real-time data and alerts for clinic staff about critical abnormalities in patients’ hydration metrics, 4) an integrated secure chat function (LINE® professional) to facilitate communication between PD patients and clinic staff, 5) integration of hydration metrics from the CKD-PD app with electronic health data in the CKDNET database on the TCC. The CKD-PD app was assessed by focus groups and usability testing processes16 and illustrated in the Multimedia Appendix1."

5-viii) Mode of delivery, features/functionalities/components of the intervention and comparator, and the theoretical framework

Describe mode of delivery, features/functionalities/components of the intervention and comparator, and the theoretical framework [6] used to design them (instructional strategy [1], behaviour change techniques, persuasive features, etc., see e.g., [7, 8] for terminology). This includes an in-depth description of the content (including where it is coming from and who developed it) [1]," whether [and how] it is tailored to individual circumstances and allows users to track their progress and receive feedback" [6]. This also includes a description of communication delivery channels and – if computer-mediated communication is a component – whether communication was synchronous or asynchronous [6]. It also includes information on presentation strategies [1], including page design principles, average amount of text on pages, presence of hyperlinks to other resources, etc. [1].

	1	2	3	4	5	
subitem not at all important	○	○	○	○	◉	essential

Clear selection

Does your paper address subitem 5-viii? *

Copy and paste relevant sections from the manuscript (include quotes in quotation marks "like this" to indicate direct quotes from your manuscript), or elaborate on this item by providing additional information not in the ms, or briefly explain why the item is not applicable/relevant for your study

"Study protocol, intervention, and definition of exposures
All participants continued standard bimonthly PD clinic visits during the study period. The study participation period was 12 months, with the actual duration varying by enrollment date, last scheduled follow-up date, or premature study termination. Participants were assigned a PD clinic visit appointment date based on their randomization allocation to reduce contamination between the two study groups. Participants in both groups used their existing home BW scales and hanging scales to weight dialysate bags to calculate UF volume and were provided with a new automatic arm BP monitor (Omron®)."

"Hydration metric collection: Hydration metric collection in both groups was performed by participants or their surrogate daily and included 1) morning BP 2) BW before first dialysis cycle 3) UF volume: difference in total weight of the dialysate fluid bags before and after each peritoneal dwell period for the preceding 24 hours. The No-App group received usual care: 1) "PD logbook"- a handwritten logbook to record hydration metrics, 2) bimonthly PD clinic appointments and 3) instructions to contact the clinic for any concerns. No other outreach was conducted unless the participant contacted the PD staff or sought care at the PD clinic or emergency department. The App group received training on how to use the CKD-PD app including hydration metric entry, self-monitoring, and in-app communication features. Participants were instructed to record their hydration metrics data daily. The PD clinic staff checked participant hydration metric data in the CKDNET database weekly. If the hydration metrics were not uploaded, the PD staff reminded the participant by telephone or chat application. Participants were provided internet access in cases of their internet signal instabilities and hydration metrics were temporarily sent via LINE® program if unexpected problems of CKD-PD app happened such as infrequent downtimes."

5-ix) Describe use parameters

Describe use parameters (e.g., intended "doses" and optimal timing for use). Clarify what instructions or recommendations were given to the user, e.g., regarding timing, frequency, heaviness of use, if any, or was the intervention used ad libitum.

	1	2	3	4	5	
subitem not at all important	○	○	○	○	●	essential

Clear selection

Does your paper address subitem 5-ix?

Copy and paste relevant sections from the manuscript (include quotes in quotation marks "like this" to indicate direct quotes from your manuscript), or elaborate on this item by providing additional information not in the ms, or briefly explain why the item is not applicable/relevant for your study

"Hydration metric collection: Hydration metric collection in both groups was performed by participants or their surrogate daily and included 1) morning BP 2) BW before first dialysis cycle 3) UF volume: difference in total weight of the dialysate fluid bags before and after each peritoneal dwell period for the preceding 24 hours. The No-App group received usual care: 1) "PD logbook"- a handwritten logbook to record hydration metrics, 2) bimonthly PD clinic appointments and 3) instructions to contact the clinic for any concerns. No other outreach was conducted unless the participant contacted the PD staff or sought care at the PD clinic or emergency department. The App group received training on how to use the CKD-PD app including hydration metric entry, self-monitoring, and in-app communication features. Participants were instructed to record their hydration metrics data daily. The PD clinic staff checked participant hydration metric data in the CKDNET database weekly. If the hydration metrics were not uploaded, the PD staff reminded the participant by telephone or chat application. Participants were provided internet access in cases of their internet signal instabilities and hydration metrics were temporarily sent via LINE® program if unexpected problems of CKD-PD app happened such as infrequent downtimes."

"Hydration metric monitoring: PD clinic staff monitored hydration metrics from the App group weekly using the CKDNET database, and during scheduled bimonthly clinic visits and unscheduled clinic contacts from both the App group and No-App group. Dry weight was individually set using the bioimpedance device together with periodically clinical adjustment by nephrologists. Hydration metrics were classified as normal, need monitoring, and action required. Criteria for an action required alert were one of more of the followings 1) deviation in BW of >3% from baseline dry weight, 2) BP>140/90 mmHg, and 3) ultrafiltration volume of < 500 ml in anuric patients. In the event of an action required alert, the PD nurse initiated contact with the participant to review hydration metrics and symptoms. If the PD nurse confirmed abnormal hydration metrics or clinical symptoms consistent with OH, the case was referred to a study nephrologist for review and clinical intervention if indicated. Clinical interventions for OH: In the No-App group, clinical interventions for OH were made at an unscheduled contact (e.g. outreach to PD clinic or emergency visit) or scheduled contact (routine clinic visit every 2 months). In the App group, clinical interventions were made when PD clinic staff contacted the patient in response to an action required alert, in addition to unscheduled and scheduled PD clinic contact. An episode of clinical intervention was defined as a treatment intervention for abnormal hydration metrics or clinical symptoms of OH and included change in antihypertension or diuretic medications, fluid or salt restriction, modification of dialysis prescription or solution, or referral for urgent visit."

5-x) Clarify the level of human involvement

Clarify the level of human involvement (care providers or health professionals, also technical assistance) in the e-intervention or as co-intervention (detail number and expertise of professionals involved, if any, as well as "type of assistance offered, the timing and frequency of the support, how it is initiated, and the medium by which the assistance is delivered". It may be necessary to distinguish between the level of human involvement required for the trial, and the level of human involvement required for a routine application outside of a RCT setting (discuss under item 21 – generalizability).

	1	2	3	4	5	
subitem not at all important	○	○	○	○	◉	essential

Clear selection

Does your paper address subitem 5-x?

Copy and paste relevant sections from the manuscript (include quotes in quotation marks "like this" to indicate direct quotes from your manuscript), or elaborate on this item by providing additional information not in the ms, or briefly explain why the item is not applicable/relevant for your study

"Hydration metric collection: Hydration metric collection in both groups was performed by participants or their surrogate daily and included 1) morning BP 2) BW before first dialysis cycle 3) UF volume: difference in total weight of the dialysate fluid bags before and after each peritoneal dwell period for the preceding 24 hours. The No-App group received usual care: 1) "PD logbook"- a handwritten logbook to record hydration metrics, 2) bimonthly PD clinic appointments and 3) instructions to contact the clinic for any concerns. No other outreach was conducted unless the participant contacted the PD staff or sought care at the PD clinic or emergency department. The App group received training on how to use the CKD-PD app including hydration metric entry, self-monitoring, and in-app communication features. Participants were instructed to record their hydration metrics data daily. The PD clinic staff checked participant hydration metric data in the CKDNET database weekly. If the hydration metrics were not uploaded, the PD staff reminded the participant by telephone or chat application. Participants were provided internet access in cases of their internet signal instabilities and hydration metrics were temporarily sent via LINE® program if unexpected problems of CKD-PD app happened such as infrequent downtimes."

"Hydration metric monitoring: PD clinic staff monitored hydration metrics from the App group weekly using the CKDNET database, and during scheduled bimonthly clinic visits and unscheduled clinic contacts from both the App group and No-App group. Dry weight was individually set using the bioimpedance device together with periodically clinical adjustment by nephrologists. Hydration metrics were classified as normal, need monitoring, and action required. Criteria for an action required alert were one of more of the followings 1) deviation in BW of >3% from baseline dry weight, 2) BP>140/90 mmHg, and 3) ultrafiltration volume of < 500 ml in anuric patients. In the event of an action required alert, the PD nurse initiated contact with the participant to review hydration metrics and symptoms. If the PD nurse confirmed abnormal hydration metrics or clinical symptoms consistent with OH, the case was referred to a study nephrologist for review and clinical intervention if indicated.
Clinical interventions for OH: In the No-App group, clinical interventions for OH were made at an unscheduled contact (e.g. outreach to PD clinic or emergency visit) or scheduled contact (routine clinic visit every 2 months). In the App group, clinical interventions were made when PD clinic staff contacted the patient in response to an action required alert, in addition to unscheduled and scheduled PD clinic contact. An episode of clinical intervention was defined as a treatment intervention for abnormal hydration metrics or clinical symptoms of OH and included change in antihypertension or diuretic medications, fluid or salt restriction, modification of dialysis prescription or solution, or referral for urgent visit."

5-xi) Report any prompts/reminders used

Report any prompts/reminders used: Clarify if there were prompts (letters, emails, phone calls, SMS) to use the application, what triggered them, frequency etc. It may be necessary to distinguish between the level of prompts/reminders required for the trial, and the level of prompts/reminders for a routine application outside of a RCT setting (discuss under item 21 – generalizability).

	1	2	3	4	5	
subitem not at all important	○	○	○	○	◉	essential

Clear selection

Does your paper address subitem 5-xi? *

Copy and paste relevant sections from the manuscript (include quotes in quotation marks "like this" to indicate direct quotes from your manuscript), or elaborate on this item by providing additional information not in the ms, or briefly explain why the item is not applicable/relevant for your study

"Hydration metric collection: Hydration metric collection in both groups was performed by participants or their surrogate daily and included 1) morning BP 2) BW before first dialysis cycle 3) UF volume: difference in total weight of the dialysate fluid bags before and after each peritoneal dwell period for the preceding 24 hours. The No-App group received usual care: 1) "PD logbook"- a handwritten logbook to record hydration metrics, 2) bimonthly PD clinic appointments and 3) instructions to contact the clinic for any concerns. No other outreach was conducted unless the participant contacted the PD staff or sought care at the PD clinic or emergency department. The App group received training on how to use the CKD-PD app including hydration metric entry, self-monitoring, and in-app communication features. Participants were instructed to record their hydration metrics data daily. The PD clinic staff checked participant hydration metric data in the CKDNET database weekly. If the hydration metrics were not uploaded, the PD staff reminded the participant by telephone or chat application. Participants were provided internet access in cases of their internet signal instabilities and hydration metrics were temporarily sent via LINE® program if unexpected problems of CKD-PD app happened such as infrequent downtimes."

"Hydration metric monitoring: PD clinic staff monitored hydration metrics from the App group weekly using the CKDNET database, and during scheduled bimonthly clinic visits and unscheduled clinic contacts from both the App group and No-App group. Dry weight was individually set using the bioimpedance device together with periodically clinical adjustment by nephrologists. Hydration metrics were classified as normal, need monitoring, and action required. Criteria for an action required alert were one of more of the followings 1) deviation in BW of >3% from baseline dry weight, 2) BP>140/90 mmHg, and 3) ultrafiltration volume of < 500 ml in anuric patients. In the event of an action required alert, the PD nurse initiated contact with the participant to review hydration metrics and symptoms. If the PD nurse confirmed abnormal hydration metrics or clinical symptoms consistent with OH, the case was referred to a study nephrologist for review and clinical intervention if indicated.
Clinical interventions for OH: In the No-App group, clinical interventions for OH were made at an unscheduled contact (e.g. outreach to PD clinic or emergency visit) or scheduled contact (routine clinic visit every 2 months). In the App group, clinical interventions were made when PD clinic staff contacted the patient in response to an action required alert, in addition to unscheduled and scheduled PD clinic contact. An episode of clinical intervention was defined as a treatment intervention for abnormal hydration metrics or clinical symptoms of OH and included change in antihypertension or diuretic medications, fluid or salt restriction, modification of dialysis prescription or solution, or referral for urgent visit."

5-xii) Describe any co-interventions (incl. training/support)

Describe any co-interventions (incl. training/support): Clearly state any interventions that are provided in addition to the targeted eHealth intervention, as ehealth intervention may not be designed as stand-alone intervention. This includes training sessions and support [1]. It may be necessary to distinguish between the level of training required for the trial, and the level of training for a routine application outside of a RCT setting (discuss under item 21 – generalizability.

	1	2	3	4	5	
subitem not at all important	○	○	◉	○	○	essential

Clear selection

Does your paper address subitem 5-xii? *

Copy and paste relevant sections from the manuscript (include quotes in quotation marks "like this" to indicate direct quotes from your manuscript), or elaborate on this item by providing additional information not in the ms, or briefly explain why the item is not applicable/relevant for your study

"Clinical interventions for OH: In the No-App group, clinical interventions for OH were made at an unscheduled contact (e.g. outreach to PD clinic or emergency visit) or scheduled contact (routine clinic visit every 2 months). In the App group, clinical interventions were made when PD clinic staff contacted the patient in response to an action required alert, in addition to unscheduled and scheduled PD clinic contact. An episode of clinical intervention was defined as a treatment intervention for abnormal hydration metrics or clinical symptoms of OH and included change in antihypertension or diuretic medications, fluid or salt restriction, modification of dialysis prescription or solution, or referral for urgent visit."

6a) Completely defined pre-specified primary and secondary outcome measures, including how and when they were assessed

Does your paper address CONSORT subitem 6a? *

Copy and paste relevant sections from the manuscript (include quotes in quotation marks "like this" to indicate direct quotes from your manuscript), or elaborate on this item by providing additional information not in the ms, or briefly explain why the item is not applicable/relevant for your study

"Data collection
Baseline data were collected from medical records and patient interviews. Hydration metrics, hydration metric monitoring, clinical symptoms, PD clinic contact and clinical interventions for OH were recorded in real time using study logs and an electronic data capture system. Outcomes of hospitalization, death and technique failure were collected at time of event notification and the hospital information system."

6a-i) Online questionnaires: describe if they were validated for online use and apply CHERRIES items to describe how the questionnaires were designed/deployed

If outcomes were obtained through online questionnaires, describe if they were validated for online use and apply CHERRIES items to describe how the questionnaires were designed/deployed [9].

	1	2	3	4	5	
subitem not at all important	○	○	◉	○	○	essential

Clear selection

Does your paper address subitem 6a-i?

Copy and paste relevant sections from manuscript text

No online questionnaires were applied in this study

6a-ii) Describe whether and how “use” (including intensity of use/dosage) was defined/measured/monitored

Describe whether and how “use” (including intensity of use/dosage) was defined/measured/monitored (logins, logfile analysis, etc.). Use/adoption metrics are important process outcomes that should be reported in any ehealth trial.

	1	2	3	4	5	
subitem not at all important	○	○	○	○	◉	essential

Clear selection

Does your paper address subitem 6a-ii?

Copy and paste relevant sections from manuscript text

"Hydration metric collection: Hydration metric collection in both groups was performed by participants or their surrogate daily and included 1) morning BP 2) BW before first dialysis cycle 3) UF volume: difference in total weight of the dialysate fluid bags before and after each peritoneal dwell period for the preceding 24 hours. The No-App group received usual care: 1) "PD logbook"- a handwritten logbook to record hydration metrics, 2) bimonthly PD clinic appointments and 3) instructions to contact the clinic for any concerns. No other outreach was conducted unless the participant contacted the PD staff or sought care at the PD clinic or emergency department. The App group received training on how to use the CKD-PD app including hydration metric entry, self-monitoring, and in-app communication features. Participants were instructed to record their hydration metrics data daily. The PD clinic staff checked participant hydration metric data in the CKDNET database weekly. If the hydration metrics were not uploaded, the PD staff reminded the participant by telephone or chat application. Participants were provided internet access in cases of their internet signal instabilities and hydration metrics were temporarily sent via LINE® program if unexpected problems of CKD-PD app happened such as infrequent downtimes."

"Hydration metric monitoring: PD clinic staff monitored hydration metrics from the App group weekly using the CKDNET database, and during scheduled bimonthly clinic visits and unscheduled clinic contacts from both the App group and No-App group. Dry weight was individually set using the bioimpedance device together with periodically clinical adjustment by nephrologists. Hydration metrics were classified as normal, need monitoring, and action required. Criteria for an action required alert were one of more of the followings 1) deviation in BW of >3% from baseline dry weight, 2) BP>140/90 mmHg, and 3) ultrafiltration volume of < 500 ml in anuric patients. In the event of an action required alert, the PD nurse initiated contact with the participant to review hydration metrics and symptoms. If the PD nurse confirmed abnormal hydration metrics or clinical symptoms consistent with OH, the case was referred to a study nephrologist for review and clinical intervention if indicated.
Clinical interventions for OH: In the No-App group, clinical interventions for OH were made at an unscheduled contact (e.g. outreach to PD clinic or emergency visit) or scheduled contact (routine clinic visit every 2 months). In the App group, clinical interventions were made when PD clinic staff contacted the patient in response to an action required alert, in addition to unscheduled and scheduled PD clinic contact. An episode of clinical intervention was defined as a treatment intervention for abnormal hydration metrics or clinical symptoms of OH and included change in antihypertension or diuretic medications, fluid or salt restriction, modification of dialysis prescription or solution, or referral for urgent visit."

6a-iii) Describe whether, how, and when qualitative feedback from participants was obtained

Describe whether, how, and when qualitative feedback from participants was obtained (e.g., through emails, feedback forms, interviews, focus groups).

	1	2	3	4	5	
subitem not at all important	○	○	○	○	◉	essential

Clear selection

Does your paper address subitem 6a-iii?

Copy and paste relevant sections from manuscript text

"4) an integrated secure chat function (LINE® professional) to facilitate communication between PD patients and clinic staff"

"The App group received training on how to use the CKD-PD app including hydration metric entry, self-monitoring, and in-app communication features. Participants were instructed to record their hydration metrics data daily. The PD clinic staff checked participant hydration metric data in the CKDNET database weekly. If the hydration metrics were not uploaded, the PD staff reminded the participant by telephone or chat application. Participants were provided internet access in cases of their internet signal instabilities and hydration metrics were temporarily sent via LINE® program if unexpected problems of CKD-PD app happened such as infrequent downtimes."

6b) Any changes to trial outcomes after the trial commenced, with reasons

Does your paper address CONSORT subitem 6b? *

Copy and paste relevant sections from the manuscript (include quotes in quotation marks "like this" to indicate direct quotes from your manuscript), or elaborate on this item by providing additional information not in the ms, or briefly explain why the item is not applicable/relevant for your study

No changes to trial outcomes after the trial commenced

7a) How sample size was determined

NPT: When applicable, details of whether and how the clustering by care provides or centers was addressed

7a-i) Describe whether and how expected attrition was taken into account when calculating the sample size

Describe whether and how expected attrition was taken into account when calculating the sample size.

	1	2	3	4	5	
subitem not at all important	○	○	○	○	◉	essential

Clear selection

Does your paper address subitem 7a-i?

Copy and paste relevant sections from manuscript title (include quotes in quotation marks "like this" to indicate direct quotes from your manuscript), or elaborate on this item by providing additional information not in the ms, or briefly explain why the item is not applicable/relevant for your study

"Sample size was calculated to determine the primary outcome by using a difference in incidence rates (IR) between two Poisson means with 25% precision. Preliminary data from Srinagarind Hospital indicated the intervention rate for OH to be 4 times/week in 91 patients or a mean intervention rate of 2.3 times/patient-year. Assuming a two-fold increase in the mean event rate for patients using the CKD-PD app or 4.6 times/patient-year in the CKD-PD app group, the desired total sample size was 80 patients for each group, using a two-sided, large-samples z-test of the Poisson event-rate difference at a significance level of 0.05. Allowing for a 10% dropout rate, a total of at least 200 patients were recruited. Recruitment targets for each facility were determined by the number of patients followed in the PD clinic: Srinagarind Hospital 20%, Khon Kaen Hospital 30%, and Chaiyaphum Hospital 50%."

7b) When applicable, explanation of any interim analyses and stopping guidelines

Does your paper address CONSORT subitem 7b? *

Copy and paste relevant sections from the manuscript (include quotes in quotation marks "like this" to indicate direct quotes from your manuscript), or elaborate on this item by providing additional information not in the ms, or briefly explain why the item is not applicable/relevant for your study

No any interim analysis was performed.

8a) Method used to generate the random allocation sequence

NPT: When applicable, how care providers were allocated to each trial group

Does your paper address CONSORT subitem 8a? *

Copy and paste relevant sections from the manuscript (include quotes in quotation marks "like this" to indicate direct quotes from your manuscript), or elaborate on this item by providing additional information not in the ms, or briefly explain why the item is not applicable/relevant for your study

"Randomization codes for the two groups were generated using computer software. Block randomization with varying block sizes of 2 and 4 was employed to ensure allocation concealment and balance between the arms. The randomization sequence was created using a random number generator and was implemented without stratification. The allocation sequence was concealed from study personnel and participants until assignment."

8b) Type of randomisation; details of any restriction (such as blocking and block size)

Does your paper address CONSORT subitem 8b? *

Copy and paste relevant sections from the manuscript (include quotes in quotation marks "like this" to indicate direct quotes from your manuscript), or elaborate on this item by providing additional information not in the ms, or briefly explain why the item is not applicable/relevant for your study

"Randomization codes for the two groups were generated using computer software. Block randomization with varying block sizes of 2 and 4 was employed to ensure allocation concealment and balance between the arms. The randomization sequence was created using a random number generator and was implemented without stratification. The allocation sequence was concealed from study personnel and participants until assignment. All enrolled participants provided written informed consent and were randomized into two equal groups at each hospital, one using the CKD-PD app (“App” group), and one receiving usual management (“No-App” group)."

9) Mechanism used to implement the random allocation sequence (such as sequentially numbered containers), describing any steps taken to conceal the sequence until interventions were assigned

Does your paper address CONSORT subitem 9? *

Copy and paste relevant sections from the manuscript (include quotes in quotation marks "like this" to indicate direct quotes from your manuscript), or elaborate on this item by providing additional information not in the ms, or briefly explain why the item is not applicable/relevant for your study

"Randomization codes for the two groups were generated by the statistician using computer software. Block randomization with varying block sizes of 2 and 4 was employed to ensure allocation concealment and balance between the arms. The randomization sequence was created using a random number generator and was implemented without stratification. The allocation sequence was concealed from study personnel and participants until assignment when research nurses enrolled and allocated participants. All enrolled participants provided written informed consent and were randomized into two equal groups at each hospital, one using the CKD-PD app (“App” group), and one receiving usual management (“No-App” group)."

10) Who generated the random allocation sequence, who enrolled participants, and who assigned participants to interventions

Does your paper address CONSORT subitem 10? *

Copy and paste relevant sections from the manuscript (include quotes in quotation marks "like this" to indicate direct quotes from your manuscript), or elaborate on this item by providing additional information not in the ms, or briefly explain why the item is not applicable/relevant for your study

"Randomization codes for the two groups were generated by the statistician using computer software. Block randomization with varying block sizes of 2 and 4 was employed to ensure allocation concealment and balance between the arms. The randomization sequence was created using a random number generator and was implemented without stratification. The allocation sequence was concealed from study personnel and participants until assignment when research nurses enrolled and allocated participants."

11a) If done, who was blinded after assignment to interventions (for example, participants, care providers, those assessing outcomes) and how

NPT: Whether or not administering co-interventions were blinded to group assignment

11a-i) Specify who was blinded, and who wasn't

Specify who was blinded, and who wasn't. Usually, in web-based trials it is not possible to blind the participants [1, 3] (this should be clearly acknowledged), but it may be possible to blind outcome assessors, those doing data analysis or those administering co-interventions (if any).

	1	2	3	4	5	
subitem not at all important	○	○	○	○	◉	essential

Clear selection

Does your paper address subitem 11a-i? *

Copy and paste relevant sections from the manuscript (include quotes in quotation marks "like this" to indicate direct quotes from your manuscript), or elaborate on this item by providing additional information not in the ms, or briefly explain why the item is not applicable/relevant for your study

The study is open-label RCT that after assignment, participants and personnel were not blinded because app usage was unable to blinded.

11a-ii) Discuss e.g., whether participants knew which intervention was the “intervention of interest” and which one was the “comparator”

Informed consent procedures (4a-ii) can create biases and certain expectations - discuss e.g., whether participants knew which intervention was the “intervention of interest” and which one was the “comparator”.

	1	2	3	4	5	
subitem not at all important	○	○	○	○	◉	essential

Clear selection

Does your paper address subitem 11a-ii?

Copy and paste relevant sections from the manuscript (include quotes in quotation marks "like this" to indicate direct quotes from your manuscript), or elaborate on this item by providing additional information not in the ms, or briefly explain why the item is not applicable/relevant for your study

"All enrolled participants provided written informed consent and were randomized into two equal groups at each hospital, one using the CKD-PD app (“App” group), and one receiving usual management (“No-App” group)."

Participants knew the CKD-PD app was the intervention of interest and usual care was the comparator.

11b) If relevant, description of the similarity of interventions

(this item is usually not relevant for ehealth trials as it refers to similarity of a placebo or sham intervention to a active medication/intervention)

Does your paper address CONSORT subitem 11b? *

Copy and paste relevant sections from the manuscript (include quotes in quotation marks "like this" to indicate direct quotes from your manuscript), or elaborate on this item by providing additional information not in the ms, or briefly explain why the item is not applicable/relevant for your study

"All participants continued standard bimonthly PD clinic visits during the study period."
"Hydration metric collection in both groups was performed by participants or their surrogate daily and included 1) morning BP 2) BW before first dialysis cycle 3) UF volume: difference in total weight of the dialysate fluid bags before and after each peritoneal dwell period for the preceding 24 hours."

"An episode of clinical intervention was defined as a treatment intervention for abnormal hydration metrics or clinical symptoms of OH and included change in antihypertension or diuretic medications, fluid or salt restriction, modification of dialysis prescription or solution, or referral for urgent visit."

12a) Statistical methods used to compare groups for primary and secondary outcomes

NPT: When applicable, details of whether and how the clustering by care providers or centers was addressed

Does your paper address CONSORT subitem 12a? *

Copy and paste relevant sections from the manuscript (include quotes in quotation marks "like this" to indicate direct quotes from your manuscript), or elaborate on this item by providing additional information not in the ms, or briefly explain why the item is not applicable/relevant for your study

"The primary outcome was presented as IR of interventions for OH and the incidence rate ratio (IRR) and its 95% confidence interval (CI) were estimated to compare both groups. The generalized estimating equation with baseline -values adjustment was performed to compare the repeated measures between the App and No-App groups. Secondary outcome for hospitalization was assessed using a multivariate Poisson regression analysis for IRR adjusted with factors that accounted for a p-value <0.05 in univariate analysis, a significant difference in baseline characteristics between the two groups, and hospital levels. Survival analysis was compared between the groups."

12a-i) Imputation techniques to deal with attrition / missing values

Imputation techniques to deal with attrition / missing values: Not all participants will use the intervention/comparator as intended and attrition is typically high in ehealth trials. Specify how participants who did not use the application or dropped out from the trial were treated in the statistical analysis (a complete case analysis is strongly discouraged, and simple imputation techniques such as LOCF may also be problematic [4]).

	1	2	3	4	5	
subitem not at all important	○	○	◉	○	○	essential

Clear selection

Does your paper address subitem 12a-i? *

Copy and paste relevant sections from the manuscript (include quotes in quotation marks "like this" to indicate direct quotes from your manuscript), or elaborate on this item by providing additional information not in the ms, or briefly explain why the item is not applicable/relevant for your study

The missing data has been found in sending information via the CKD-PD App, however, hydration metric upload compliance in the App group was acceptable (85.7 %).

12b) Methods for additional analyses, such as subgroup analyses and adjusted analyses

Does your paper address CONSORT subitem 12b? *

Copy and paste relevant sections from the manuscript (include quotes in quotation marks "like this" to indicate direct quotes from your manuscript), or elaborate on this item by providing additional information not in the ms, or briefly explain why the item is not applicable/relevant for your study

"Secondary outcome for hospitalization was assessed using a multivariate Poisson regression analysis for IRR adjusted with factors that accounted for a p-value <0.05 in univariate analysis, a significant difference in baseline characteristics between the two groups, and hospital levels."

X26) REB/IRB Approval and Ethical Considerations [recommended as subheading under "Methods"] (not a CONSORT item)

X26-i) Comment on ethics committee approval

	1	2	3	4	5	
subitem not at all important	○	○	○	○	◉	essential

Clear selection

Does your paper address subitem X26-i?

Copy and paste relevant sections from the manuscript (include quotes in quotation marks "like this" to indicate direct quotes from your manuscript), or elaborate on this item by providing additional information not in the ms, or briefly explain why the item is not applicable/relevant for your study

"The study protocol was approved by the Khon Kaen University Ethics Committee for Human Research, (HE 621494) in accordance with the ethical principles of the Declaration of Helsinki, the Good Clinical Practice guidelines"

x26-ii) Outline informed consent procedures

Outline informed consent procedures e.g., if consent was obtained offline or online (how? Checkbox, etc.?), and what information was provided (see 4a-ii). See [6] for some items to be included in informed consent documents.

	1	2	3	4	5	
subitem not at all important	○	○	○	○	◉	essential

Clear selection

Does your paper address subitem X26-ii?

Copy and paste relevant sections from the manuscript (include quotes in quotation marks "like this" to indicate direct quotes from your manuscript), or elaborate on this item by providing additional information not in the ms, or briefly explain why the item is not applicable/relevant for your study

"Informed consent was obtained offline and was shown as the supplementary material."

X26-iii) Safety and security procedures

Safety and security procedures, incl. privacy considerations, and any steps taken to reduce the likelihood or detection of harm (e.g., education and training, availability of a hotline)

	1	2	3	4	5	
subitem not at all important	○	○	◉	○	○	essential

Clear selection

Does your paper address subitem X26-iii?

Copy and paste relevant sections from the manuscript (include quotes in quotation marks "like this" to indicate direct quotes from your manuscript), or elaborate on this item by providing additional information not in the ms, or briefly explain why the item is not applicable/relevant for your study

No relevant of harm procedure from app intervention.

RESULTS

13a) For each group, the numbers of participants who were randomly assigned, received intended treatment, and were analysed for the primary outcome

NPT: The number of care providers or centers performing the intervention in each group and the number of patients treated by each care provider in each center

Does your paper address CONSORT subitem 13a? *

Copy and paste relevant sections from the manuscript (include quotes in quotation marks "like this" to indicate direct quotes from your manuscript), or elaborate on this item by providing additional information not in the ms, or briefly explain why the item is not applicable/relevant for your study

"The outcomes were analyzed as intent-to-treat."
"A total of 208 participants were randomized into the 2 groups at each study site resulting in 103 subjects in the App group and 105 cases in the No-App group (Table 1)."

13b) For each group, losses and exclusions after randomisation, together with reasons

Does your paper address CONSORT subitem 13b? (NOTE: Preferably, this is shown in a CONSORT flow diagram) *

Copy and paste relevant sections from the manuscript (include quotes in quotation marks "like this" to indicate direct quotes from your manuscript), or elaborate on this item by providing additional information not in the ms, or briefly explain why the item is not applicable/relevant for your study

"There were 23 participants who were prematurely terminated in the App group and 30 in the No-app group. At the end of study, 74.8 % of the App group and 69.5% of the No-App group completed the entire follow-up period as shown in the study flow diagram (Figure 1)."

13b-i) Attrition diagram

Strongly recommended: An attrition diagram (e.g., proportion of participants still logging in or using the intervention/comparator in each group plotted over time, similar to a survival curve) or other figures or tables demonstrating usage/dose/engagement.

	1	2	3	4	5	
subitem not at all important	○	○	○	○	◉	essential

Clear selection

Does your paper address subitem 13b-i?

Copy and paste relevant sections from the manuscript or cite the figure number if applicable (include quotes in quotation marks "like this" to indicate direct quotes from your manuscript), or elaborate on this item by providing additional information not in the ms, or briefly explain why the item is not applicable/relevant for your study

The data are shown in Figure 1

14a) Dates defining the periods of recruitment and follow-up

Does your paper address CONSORT subitem 14a? *

Copy and paste relevant sections from the manuscript (include quotes in quotation marks "like this" to indicate direct quotes from your manuscript), or elaborate on this item by providing additional information not in the ms, or briefly explain why the item is not applicable/relevant for your study

"This study was an open-label randomized control trial conducted between December 2021 and February 2023 "
"The median participants follow-up times were similar between the App [11.2 (9.4 – 12.0) months] and No-App groups [11.0 (9.4 – 11.9) months], p=0.49."

14a-i) Indicate if critical "secular events" fell into the study period

Indicate if critical "secular events" fell into the study period, e.g., significant changes in Internet resources available or "changes in computer hardware or Internet delivery resources"

	1	2	3	4	5	
subitem not at all important	○	○	◉	○	○	essential

Clear selection

Does your paper address subitem 14a-i?

Copy and paste relevant sections from the manuscript (include quotes in quotation marks "like this" to indicate direct quotes from your manuscript), or elaborate on this item by providing additional information not in the ms, or briefly explain why the item is not applicable/relevant for your study

"Participants were provided internet access in cases of their internet signal instabilities and hydration metrics were temporarily sent via LINE® program if unexpected problems of CKD-PD app happened such as infrequent downtimes."

14b) Why the trial ended or was stopped (early)

Does your paper address CONSORT subitem 14b? *

Copy and paste relevant sections from the manuscript (include quotes in quotation marks "like this" to indicate direct quotes from your manuscript), or elaborate on this item by providing additional information not in the ms, or briefly explain why the item is not applicable/relevant for your study

The trial was not early stopped.

15) A table showing baseline demographic and clinical characteristics for each group

NPT: When applicable, a description of care providers (case volume, qualification, expertise, etc.) and centers (volume) in each group

Does your paper address CONSORT subitem 15? *

Copy and paste relevant sections from the manuscript (include quotes in quotation marks "like this" to indicate direct quotes from your manuscript), or elaborate on this item by providing additional information not in the ms, or briefly explain why the item is not applicable/relevant for your study

The data are presented in Table 1

15-i) Report demographics associated with digital divide issues

In ehealth trials it is particularly important to report demographics associated with digital divide issues, such as age, education, gender, social-economic status, computer/Internet/ehealth literacy of the participants, if known.

	1	2	3	4	5	
subitem not at all important	○	○	○	○	◉	essential

Clear selection

Does your paper address subitem 15-i? *

Copy and paste relevant sections from the manuscript (include quotes in quotation marks "like this" to indicate direct quotes from your manuscript), or elaborate on this item by providing additional information not in the ms, or briefly explain why the item is not applicable/relevant for your study

They are presented in Table 1.

16) For each group, number of participants (denominator) included in each analysis and whether the analysis was by original assigned groups

16-i) Report multiple "denominators" and provide definitions

Report multiple "denominators" and provide definitions: Report N's (and effect sizes) "across a range of study participation [and use] thresholds" [1], e.g., N exposed, N consented, N used more than x times, N used more than y weeks, N participants "used" the intervention/comparator at specific pre-defined time points of interest (in absolute and relative numbers per group). Always clearly define "use" of the intervention.

	1	2	3	4	5	
subitem not at all important	○	○	○	○	◉	essential

Clear selection

Does your paper address subitem 16-i? *

Copy and paste relevant sections from the manuscript (include quotes in quotation marks "like this" to indicate direct quotes from your manuscript), or elaborate on this item by providing additional information not in the ms, or briefly explain why the item is not applicable/relevant for your study

The data are revealed in Table 2 and 3.

16-ii) Primary analysis should be intent-to-treat

Primary analysis should be intent-to-treat, secondary analyses could include comparing only “users”, with the appropriate caveats that this is no longer a randomized sample (see 18-i).

	1	2	3	4	5	
subitem not at all important	○	○	○	○	◉	essential

Clear selection

Does your paper address subitem 16-ii?

Copy and paste relevant sections from the manuscript (include quotes in quotation marks "like this" to indicate direct quotes from your manuscript), or elaborate on this item by providing additional information not in the ms, or briefly explain why the item is not applicable/relevant for your study

"The outcomes were analyzed as intent-to-treat."

17a) For each primary and secondary outcome, results for each group, and the estimated effect size and its precision (such as 95% confidence interval)

Does your paper address CONSORT subitem 17a? *

Copy and paste relevant sections from the manuscript (include quotes in quotation marks "like this" to indicate direct quotes from your manuscript), or elaborate on this item by providing additional information not in the ms, or briefly explain why the item is not applicable/relevant for your study

"The IR of overall clinical intervention was 2.5 times significantly higher in the App group, i.e., IRR 0.4 (95% CI 0.35 - 0.46, p< 0.001) if the App group was reference. "

17a-i) Presentation of process outcomes such as metrics of use and intensity of use

In addition to primary/secondary (clinical) outcomes, the presentation of process outcomes such as metrics of use and intensity of use (dose, exposure) and their operational definitions is critical. This does not only refer to metrics of attrition (13-b) (often a binary variable), but also to more continuous exposure metrics such as “average session length”. These must be accompanied by a technical description how a metric like a “session” is defined (e.g., timeout after idle time) [1] (report under item 6a).

	1	2	3	4	5	
subitem not at all important	○	○	◉	○	○	essential

Clear selection

Does your paper address subitem 17a-i?

Copy and paste relevant sections from the manuscript (include quotes in quotation marks "like this" to indicate direct quotes from your manuscript), or elaborate on this item by providing additional information not in the ms, or briefly explain why the item is not applicable/relevant for your study

"Hydration metric upload compliance in the App group was 85.7 % (71.4 - 95.6) with the highest rate by participants at Khon Kaen Hospital (91.8%). Total follow-up visits in the App group were 3,657 with 3,145 using the CKD-PD app and 512 at scheduled PD clinic appointment."

17b) For binary outcomes, presentation of both absolute and relative effect sizes is recommended

Does your paper address CONSORT subitem 17b? *

Copy and paste relevant sections from the manuscript (include quotes in quotation marks "like this" to indicate direct quotes from your manuscript), or elaborate on this item by providing additional information not in the ms, or briefly explain why the item is not applicable/relevant for your study

The data are shown in Table 4-6.

18) Results of any other analyses performed, including subgroup analyses and adjusted analyses, distinguishing pre-specified from exploratory

Does your paper address CONSORT subitem 18? *

Copy and paste relevant sections from the manuscript (include quotes in quotation marks "like this" to indicate direct quotes from your manuscript), or elaborate on this item by providing additional information not in the ms, or briefly explain why the item is not applicable/relevant for your study

The data are presented in Supplementary Tables.

18-i) Subgroup analysis of comparing only users

A subgroup analysis of comparing only users is not uncommon in ehealth trials, but if done, it must be stressed that this is a self-selected sample and no longer an unbiased sample from a randomized trial (see 16-iii).

	1	2	3	4	5	
subitem not at all important	(selected)	○	○	○	○	essential

Clear selection

Does your paper address subitem 18-i?

Copy and paste relevant sections from the manuscript (include quotes in quotation marks "like this" to indicate direct quotes from your manuscript), or elaborate on this item by providing additional information not in the ms, or briefly explain why the item is not applicable/relevant for your study

No relevance in this study

19) All important harms or unintended effects in each group
(for specific guidance see CONSORT for harms)

Does your paper address CONSORT subitem 19? *

Copy and paste relevant sections from the manuscript (include quotes in quotation marks "like this" to indicate direct quotes from your manuscript), or elaborate on this item by providing additional information not in the ms, or briefly explain why the item is not applicable/relevant for your study

No important harms in both groups

19-i) Include privacy breaches, technical problems

Include privacy breaches, technical problems. This does not only include physical “harm” to participants, but also incidents such as perceived or real privacy breaches [1], technical problems, and other unexpected/unintended incidents. “Unintended effects” also includes unintended positive effects [2].

	1	2	3	4	5	
subitem not at all important	○	○	◉	○	○	essential

Clear selection

Does your paper address subitem 19-i?

Copy and paste relevant sections from the manuscript (include quotes in quotation marks "like this" to indicate direct quotes from your manuscript), or elaborate on this item by providing additional information not in the ms, or briefly explain why the item is not applicable/relevant for your study

The users had allowed and consented for sending their health data.
No privacy breaches occurred with this study

19-ii) Include qualitative feedback from participants or observations from staff/researchers

Include qualitative feedback from participants or observations from staff/researchers, if available, on strengths and shortcomings of the application, especially if they point to unintended/unexpected effects or uses. This includes (if available) reasons for why people did or did not use the application as intended by the developers.

	1	2	3	4	5	
subitem not at all important	○	○	◉	○	○	essential

Clear selection

Does your paper address subitem 19-ii?

Copy and paste relevant sections from the manuscript (include quotes in quotation marks "like this" to indicate direct quotes from your manuscript), or elaborate on this item by providing additional information not in the ms, or briefly explain why the item is not applicable/relevant for your study

The feedback has been reported from our previous study (reference no. 16) and we have improved the App for more convenience in using.

DISCUSSION

22) Interpretation consistent with results, balancing benefits and harms, and considering other relevant evidence

NPT: In addition, take into account the choice of the comparator, lack of or partial blinding, and unequal expertise of care providers or centers in each group

22-i) Restate study questions and summarize the answers suggested by the data, starting with primary outcomes and process outcomes (use)

Restate study questions and summarize the answers suggested by the data, starting with primary outcomes and process outcomes (use).

	1	2	3	4	5	
subitem not at all important	○	○	○	○	◉	essential

Clear selection

Does your paper address subitem 22-i? *

Copy and paste relevant sections from the manuscript (include quotes in quotation marks "like this" to indicate direct quotes from your manuscript), or elaborate on this item by providing additional information not in the ms, or briefly explain why the item is not applicable/relevant for your study

"This is the first randomized controlled trial in a middle income country to demonstrate that use of a mobile health application (CKD-PD) can improve detection and treatment of OH in PD patients resulting in improved BP control, less weight gain, less OH severity, and decrease in hospitalizations from all cause and volume overload."

22-ii) Highlight unanswered new questions, suggest future research

Highlight unanswered new questions, suggest future research.

	1	2	3	4	5	
subitem not at all important	○	○	○	○	◉	essential

Clear selection

Does your paper address subitem 22-ii?

Copy and paste relevant sections from the manuscript (include quotes in quotation marks "like this" to indicate direct quotes from your manuscript), or elaborate on this item by providing additional information not in the ms, or briefly explain why the item is not applicable/relevant for your study

"A larger RCT with more participants at different types of study sites is needed to provide robust evidence on mortality. Additional research into the cost effectiveness is another important study for direct resources and policy regarding the use of mobile health technology and RPM for PD patients."

20) Trial limitations, addressing sources of potential bias, imprecision, and, if relevant, multiplicity of analyses

20-i) Typical limitations in ehealth trials

Typical limitations in ehealth trials: Participants in ehealth trials are rarely blinded. Ehealth trials often look at a multiplicity of outcomes, increasing risk for a Type I error. Discuss biases due to non-use of the intervention/usability issues, biases through informed consent procedures, unexpected events.

	1	2	3	4	5	
subitem not at all important	○	○	○	○	◉	essential

Clear selection

Does your paper address subitem 20-i? *

Copy and paste relevant sections from the manuscript (include quotes in quotation marks "like this" to indicate direct quotes from your manuscript), or elaborate on this item by providing additional information not in the ms, or briefly explain why the item is not applicable/relevant for your study

"There are some limitations to this study. During our enrollment, the Thai government changed the PD First policy, allowing PD patients to choose between PD and hemodialysis. As a result, some participants withdrew early, impacting our sample size. The number of PD patients at each of the hospital sites was not enough to stratify and compare patients from different settings for all outcomes. The median follow-up duration was 11.2 months with 70– 75% of subjects completing the entire study, therefore premature termination might affect our results."

21) Generalisability (external validity, applicability) of the trial findings

NPT: External validity of the trial findings according to the intervention, comparators, patients, and care providers or centers involved in the trial

21-i) Generalizability to other populations

Generalizability to other populations: In particular, discuss generalizability to a general Internet population, outside of a RCT setting, and general patient population, including applicability of the study results for other organizations

	1	2	3	4	5	
subitem not at all important	○	○	○	○	◉	essential

Clear selection

Does your paper address subitem 21-i?

Copy and paste relevant sections from the manuscript (include quotes in quotation marks "like this" to indicate direct quotes from your manuscript), or elaborate on this item by providing additional information not in the ms, or briefly explain why the item is not applicable/relevant for your study

"These findings suggest that App users at all three sites benefitted from more frequent monitoring of hydration status and improved opportunity for early intervention regardless of hospital level and location"

21-ii) Discuss if there were elements in the RCT that would be different in a routine application setting

Discuss if there were elements in the RCT that would be different in a routine application setting (e.g., prompts/reminders, more human involvement, training sessions or other co-interventions) and what impact the omission of these elements could have on use, adoption, or outcomes if the intervention is applied outside of a RCT setting.

	1	2	3	4	5	
subitem not at all important	○	○	○	○	◉	essential

Clear selection

Does your paper address subitem 21-ii?

Copy and paste relevant sections from the manuscript (include quotes in quotation marks "like this" to indicate direct quotes from your manuscript), or elaborate on this item by providing additional information not in the ms, or briefly explain why the item is not applicable/relevant for your study

"Most OH events in the App group were managed through salt and water restriction along with anti-hypertensive medication adjustments - simpler, lower risk and less expensive interventions - compared with than those in the No-app group which had more severe signs and symptoms of OH and required greater percentage of complex interventions such as diuretics, hypertonic peritoneal solution administration and change of PD prescription."

OTHER INFORMATION

23) Registration number and name of trial registry

Does your paper address CONSORT subitem 23? *

Copy and paste relevant sections from the manuscript (include quotes in quotation marks "like this" to indicate direct quotes from your manuscript), or elaborate on this item by providing additional information not in the ms, or briefly explain why the item is not applicable/relevant for your study

"The study was registered in the Clinical Trials Registry (Clinicaltrials.gov ID#NCT04797195 with the first postdate 15/03/2021)."

24) Where the full trial protocol can be accessed, if available

Does your paper address CONSORT subitem 24? *

Cite a Multimedia Appendix, other reference, or copy and paste relevant sections from the manuscript (include quotes in quotation marks "like this" to indicate direct quotes from your manuscript), or elaborate on this item by providing additional information not in the ms, or briefly explain why the item is not applicable/relevant for your study

it can be accessed in the Clinicaltrials.gov ID#NCT04797195.

25) Sources of funding and other support (such as supply of drugs), role of funders

Does your paper address CONSORT subitem 25? *

Copy and paste relevant sections from the manuscript (include quotes in quotation marks "like this" to indicate direct quotes from your manuscript), or elaborate on this item by providing additional information not in the ms, or briefly explain why the item is not applicable/relevant for your study

"The research reported in this publication was supported by the Fogarty International Center of the National Institutes of Health under Award Number R21TW010963 and the CKDNET project. The content is solely the responsibility of the authors and does not necessarily represent the official views of the National Institutes of Health."

X27) Conflicts of Interest (not a CONSORT item)

X27-i) State the relation of the study team towards the system being evaluated

In addition to the usual declaration of interests (financial or otherwise), also state the relation of the study team towards the system being evaluated, i.e., state if the authors/evaluators are distinct from or identical with the developers/sponsors of the intervention.

	1	2	3	4	5	
subitem not at all important	○	○	○	○	◉	essential

Clear selection

Does your paper address subitem X27-i?

Copy and paste relevant sections from the manuscript (include quotes in quotation marks "like this" to indicate direct quotes from your manuscript), or elaborate on this item by providing additional information not in the ms, or briefly explain why the item is not applicable/relevant for your study

"Bandit Thinkhamrop is one of the developers of CKD-PD app, however, he was not involved in the process of methodology such as data curation, investigation, collection, and statistical analysis."

About the CONSORT EHEALTH checklist

As a result of using this checklist, did you make changes in your manuscript? *

- ○ yes, major changes
- ◉ yes, minor changes
- ○ no

What were the most important changes you made as a result of using this checklist?

Description of more details about the CKD-PD App

How much time did you spend on going through the checklist INCLUDING making changes in your manuscript *

1-2 hours per day for 1 week

As a result of using this checklist, do you think your manuscript has improved? *

- (x) yes
- ( ) no
- ( ) Other:

Would you like to become involved in the CONSORT EHEALTH group?

This would involve for example becoming involved in participating in a workshop and writing an "Explanation and Elaboration" document

- ( ) yes
- ( ) no
- (x) Other: I prefer if I have a free time

Clear selection

Any other comments or questions on CONSORT EHEALTH

Thank you for this valuable CONSORT EHEALTH

STOP - Save this form as PDF before you click submit

To generate a record that you filled in this form, we recommend to generate a PDF of this page (on a Mac, simply select "print" and then select "print as PDF") before you submit it.

When you submit your (revised) paper to JMIR, please upload the PDF as supplementary file.

Don't worry if some text in the textboxes is cut off, as we still have the complete information in our database. Thank you!

Final step: Click submit !

Click submit so we have your answers in our database!

Submit Clear form

Never submit passwords through Google Forms.

This form was created outside of your domain. - Terms of Service - Privacy Policy

Does this form look suspicious? Report

Google Forms
