# Supplementary material for: Effect of the Chronic Kidney Disease—Peritoneal Dialysis (CKD-PD) App on Improvement of Overhydration Treatment in Patients on Peritoneal Dialysis: Randomized Controlled Trial
Source: J Med Internet Res. 2025 May 21;27:e70641. doi: 10.2196/70641 (PMC12138318; doi:10.2196/70641)
Supplement: Multimedia Appendix 3 [file jmir_v27i1e70641_app3.pdf]

Multimedia Appendix 3. Baseline characteristics of participants at the three hospitals

**Characteristics**	**Total (n=208)**	**Srinagarind Hospital (n=40)**	**Khon Kaen Hospital (n=72)**	**Chaiyaphum Hospital (n=96)**	***P* Value**
Age (years), mean ± SD*	54.3 ± 15.0	60.9 ± 11.7	50.3 ± 15.6	54.6 ± 14.8	.001
Sex, n (%)					.13
Male	91 (43.8)	23 (57.5)	31 (43.1)	37 (38.5)	
Female	117 (56.2)	17 (42.5)	41 (56.9)	59 (61.5)	
Educational levels, n (%)*					<.001
Less than high school	128 (61.5)	16 (40.0)	50 (69.4)	62 (64.6)	
High school	47 (22.6)	7 (17.5)	14 (19.4)	26 (27.1)	
Occupational program	9 (4.3)	2 (5.0)	5 (6.9)	2 (2.1)	
Bachelor's degree	19 (9.1)	11 (27.5)	3 (4.2)	5 (5.2)	
More than Bachelor's degree	5 (2.4)	4 (10.0)	0 (0.0)	1 (1.0)	
Distance from PD clinic (km.), median (IQR)	41 (15 – 80)	38 (11 – 100)	35 (20 - 57.5)	45 (30 – 100)	.29
Cause of CKD, n (%)*					<.001
Diabetes Mellitus	96 (46.2)	22 (55.0)	28 (38.9)	46 (47.9)	
Hypertension	47 (22.6)	8 (20.0)	33 (45.8)	6 (6.3)	
Glomerulonephritis	11 (5.3)	4 (10.0)	3 (4.2)	4 (4.2)	
Gout	4 (1.9)	1 (2.5)	2 (2.8)	1 (1.0)	
Others	11 (5.3)	2 (5.0)	2 (2.8)	7 (7.3)	
Unknown	39 (18.8)	3 (7.5)	4 (5.6)	32 (33.3)	
Comorbidities, n (%)*					<.001
None	12 (5.8)	0 (0.0)	1 (1.4)	11 (11.5)	
Hypertension	155 (74.5)	39 (97.5)	60 (83.3)	56 (58.3)	
Dyslipidemia	41 (19.7)	30 (75.0)	7 (9.7)	4 (4.5)	
Coronary heart disease	11 (5.3)	7 (17.0)	3 (4.2)	1 (1.0)	
Cerebrovascular disease	8 (3.8)	5 (12.0)	3 (4.2)	0 (0.0)	
Cancers	1 (0.5)	1 (2.5)	0 (0.0)	0 (0.0)	
Others	44 (21.2)	13 (32.5)	16 (22.2)	15 (15.63)	
Smoking history status, n (%)*					.005
Never smoked	152 (73.1)	28 (70.0)	45 (62.5)	79 (82.3)	
Ex-smoked	53 (25.5)	10 (25.0)	27 (37.5)	16 (16.7)	
Current smoked	3 (1.4)	2 (5.0)	0 (0.0)	1 (1.0)	
Alcohol History status, n (%)*					<.001
Never drunk	143 (68.8)	26 (65.0)	33 (45.8)	84 (87.5)	
Ex- drunk	64 (30.8)	14 (35.0)	39 (54.2)	11 (11.5)	
Current drunk	1 (0.5)	0 (0.0)	0 (0.0)	1 (1.0)	
Clinical parameters					
Body weight (kg), mean ± SD	58.7 ± 13.2	60.7 ± 12.8	59.7 ± 15.9	57.1 ± 10.8	.26
Height (cm.), mean ± SD	159.9 ± 8.7	160.8 ± 9.3	160.4 ± 8.3	159.1 ± 8.8	.47
BMI (kg/m^2), mean ± SD	22.8 ± 4.1	23.4 ± 3.9	23.1 ± 5.1	22.5 ± 3.3	.45
SBP (mmHg), mean ± SD	148.2 ± 26.1	149.0 ± 26.7	144.4 ± 20.8	150.8 ± 29.2	.29
DBP (mmHg), mean ± SD*	81.1 ± 18.8	74.3 ± 18.5	80.0 ± 15.4	84.7 ± 20.5	.01
Daily urine (mL/day), median (IQR)*	300 (50 - 664)	382 (50 - 800)	150 (0 - 400)	442 (100 -775)	.01
Clinical signs of hypervolemia, n (%)					
Orthopnea	2 (1.0)	1 (2.5)	1 (1.4)	0 (0.0)	.29
PND / DOE*	2 (1.0)	2 (5.0)	0 (0.0)	0 (0.0)	.04
Hypertension (BP >140/90 mmHg)*	65 (31.3)	6 (15.0)	22 (30.6)	37 (38.5)	.03
Edema*	47 (22.6)	18 (45.0)	9 (12.5)	20 (20.8)	<.001
Medications					
Furosemide, n (%)*	123 (59.1)	18 (45.0)	39 (54.2)	66 (68.8)	.03
Antihypertensive drugs, n (%)*	182 (87.5)	38 (95.0)	66 (91.7)	78 (81.3)	.04
ESA, n (%)*	190 (91.3)	32 (80.0)	68 (94.4)	90 (93.8)	.03

Characteristics	Total (n=208)	Srinagarind Hospital (n=40)	Khon Kaen Hospital (n=72)	Chaiyaphum Hospital (n=96)	P Value
Laboratory profiles, mean ± SD					
Hemoglobin (g/dL)	9.9 ± 1.9	10.2 ± 1.7	9.8 ± 1.9	9.9 ± 1.9	.54
Hematocrit (%)	30.6 ± 5.8	31.8± 5.6	30.4 ± 5.8	30.4 ± 5.9	.39
Fasting Blood Sugar (mg/dL)**	131.8 ± 60.3	127.7 ± 53.4	136.7 ± 57.8	130.9 ± 66.1	.80
Blood Urea Nitrogen (mg/dL)	53.3 ± 19.2	55.2 ± 14.1	50.6 ± 21.9	54.5 ± 18.9	.33
Creatinine (mg/dL)	10.3 ± 4.4	9.4 ± 4.1	10.8 ± 4.0	10.3 ± 4.6	.24
Sodium (mEq/L)	135.5 ± 5.2	136.8 ± 5.2	135.6 ± 4.8	134.9 ± 5.4	.17
Potassium (mEq/L)*	4.1 ± 0.8	4.4 ± 0.7	3.8 ± 0.7	4.1 ± 0.8	<.001
Bicarbonate (mEq/L)*	27.2 ± 4.2	25.2 ± 2.9	26.1 ± 3.9	28.9 ± 4.3	<.001
Chloride (mEq/L)	94.8 ± 9.8	96.6 ± 13.8	95.0 ± 11.4	93.7 ± 5.5	.28
Calcium (mg/dL)*	8.4 ± 1.0	8.9 ± 0.9	8.4 ± 0.9	8.2 ± 1.1	<.001
Phosphorus (mg/dL)*	4.5 ± 1.8	4.4 ± 1.3	3.9 ± 1.5	4.9 ± 2.0	.004
Albumin (g/dL)*	3.3 ± 0.6	3.5 ± 0.5	3.5 ± 0.7	3.0 ± 0.5	<.001
Parathyroid hormone (pg/mL)*	279 (153- 473)	184 (88-355)	359 (231-682)	263 (156-428)	.001
PD information					
Type of TK catheter, %*					<.001
Straight / curl	16.3 / 83.7	57.5 / 42.5	15.3 / 84.7	0.00 / 100.00	
Incision site, %*					<.001
Midline / paramedian	79.8 / 20.2	2.5 / 97.5	98.6 / 1.4	97.9 / 2.1	
Mode of PD, n (%)*					<.001
CAPD	109 (52.4)	17 (42.5)	72 (100)	20 (20.8)	
DAPD	70 (33.7)	0 (0.0)	0 (0.0)	70 (72.9)	
NIPD	26 (12.5)	21 (52.5)	0 (0.0)	5 (5.2)	
CCPD	3 (1.4)	2 (5.0)	0 (0.0)	1 (1.0)	
Types of peritoneal membrane transport, %					.79
Fast/ average/ slow	6.2 / 85.6 / 8.2	10.0 / 80.0 / 10.0	4.2 / 87.5 / 8.3	5.2 / 87.5 / 7.3	
PD exchanger, %					.30
Patient / family member	54.8 / 45.2	55.0 / 45.0	61.1 / 38.9	49.0 / 51.0	
PD solution, n (%)					
1.5 % dextrose*	206 (99.0)	38 (95.0)	72 (100.0)	96 (100.0)	.04
2.5 % dextrose	19 (9.1)	1 (2.5)	8 (11.1)	10 (10.4)	.29
4.25 % dextrose	16 (7.7)	0 (0.0)	7 (9.7)	9 (9.4)	.09
7.5 % icodextrin*	2 (1.0)	2 (5.0)	0 (0.0)	0 (0.0)	.04
Daily UF (mL/day), median (IQR)	700 (450-1000)	600 (407-1000)	783 (533-1000)	650 (400-920)	.43
History of peritonitis, n (%)*	46 (22.1)	12 (30.0)	24 (33.3)	10 (10.4)	.001
History of exit site infection, n (%)*	30 (14.4)	12 (30.0)	8 (11.1)	10 (10.4)	.008
History of catheter malfunction, n (%)*	14 (6.7)	7 (17.5)	6 (8.3)	1 (1.0)	.001

***** p-value < 0.05, ** Tested in diabetic patients; CKD, chronic kidney disease; BMI, body mass index; PND, paroxysmal nocturnal dyspnea; DOE, dyspnea on exertion; SBP, systolic blood pressure; DBP, diastolic blood pressure; ESA, erythropoiesis-stimulating agent; CAPD, continuous ambulatory peritoneal dialysis; DAPD, daytime ambulatory peritoneal dialysis; NIPD, nocturnal intermittent peritoneal dialysis; CCPD, continuous cycling peritoneal dialysis; UF, ultrafiltration; n, number; SD, standard deviation; IQR, interquartile range
