# Supplementary material for: Effect of the Chronic Kidney Disease—Peritoneal Dialysis (CKD-PD) App on Improvement of Overhydration Treatment in Patients on Peritoneal Dialysis: Randomized Controlled Trial
Source: J Med Internet Res. 2025 May 21;27:e70641. doi: 10.2196/70641 (PMC12138318; doi:10.2196/70641)
Supplement: Multimedia Appendix 4 [file jmir_v27i1e70641_app4.pdf]

Multimedia Appendix 4. Incidence rate ratio of interventions for overhydration between the App and No-App groups at the three hospitals

Hospitals	Overall number	Incidence rate*		IRR compared between the App and No-App				IRR compared among the 3 hospitals		
		App	No-App	App	No-App	95% CI	*P*-Value	IRR	95 % CI	*P*-Value
		**All clinical interventions for overhydration**								
Srinagarind	40	177.10	45.07	1	0.25	0.20 – 0.32	<0.001	1		
Khon Kaen	72	67.58	40.67	1	0.60	0.49 – 0.74	<0.001	0.47	0.41 – 0.53	<.001
Chaiyaphum	96	21.41	7.02	1	0.33	0.22 - 0.49	<0.001	0.13	0.11 – 0.15	<.001
		**Advice of dietary change**								
Srinagarind	40	87.33	19.00	1	0.22	0.15 – 0.31	<0.001	1		
Khon Kaen	72	54.89	24.50	1	0.45	0.35 – 0.57	<0.001	0.71	0.59 – 0.84	<.001
Chaiyaphum	96	14.54	2.94	1	0.20	0.11 - 0.37	<0.001	0.17	0.13 – 0.21	<.001
		**Prescription of hypertensive drugs**								
Srinagarind	40	47.59	8.69	1	0.18	0.11 – 0.31	<0.001	1		
Khon Kaen	72	0.59	0.26	1	0.44	0.04 – 4.87	0.51	0.01	0.005 – 0.04	<.001
Chaiyaphum	96	2.22	1.36	1	0.61	0.23 – 1.65	0.33	0.06	0.04 – 0.10	<.001
		**Prescription of diuretic drugs**								
Srinagarind	40	11.28	8.69	1	0.77	0.41 – 1.46	0.42	1		
Khon Kaen	72	2.36	2.35	1	0.99	0.38 – 2.58	0.99	0.23	0.13 – 0.41	<.001
Chaiyaphum	96	0.20	0.00	1	NA	NA	NA	0.01	0.001 – 0.08	<.001
		**Prescription of hypertonic solution**								
Srinagarind	40	20.60	5.43	1	0.26	0.13 – 0.53	<0.001	1		
Khon Kaen	72	8.56	10.43	1	1.22	0.76 – 1.97	0.42	0.71	0.50 – 1.02	.065
Chaiyaphum	96	1.62	1.13	1	0.70	0.23 – 2.14	0.53	0.10	0.06 – 0.19	<.001
		**Change of PD prescription**								
Srinagarind	40	8.34	3.26	1	0.39	0.15 – 0.99	0.048	1		
Khon Kaen	72	1.18	3.13	1	2.65	0.85 – 8.22	0.09	0.37	0.20 – 0.71	.002
Chaiyaphum	96	2.83	1.58	1	0.56	0.23 – 1.39	0.21	0.38	0.21 – 0.68	.001
		**Prescription of 7.5% icodextrin**								
Srinagarind	40	1.96	0.00	1	NA	NA	NA	NA	NA	NA
Khon Kaen	72	0.00	0.00	1	NA	NA	NA	NA	NA	NA
Chaiyaphum	96	0.00	0.00	1	NA	NA	NA	NA	NA	NA

* events/100 person-month; IRR, incidence rate ratio; CI, confidence interval; App, application; NA, non-applicable
