# Supplementary material for: Effect of the Chronic Kidney Disease—Peritoneal Dialysis (CKD-PD) App on Improvement of Overhydration Treatment in Patients on Peritoneal Dialysis: Randomized Controlled Trial
Source: J Med Internet Res. 2025 May 21;27:e70641. doi: 10.2196/70641 (PMC12138318; doi:10.2196/70641)
Supplement: Multimedia Appendix 5 [file jmir_v27i1e70641_app5.pdf]

Multimedia Appendix 5. Laboratory profiles at the last hospital visits of participants

Characteristics	Total *(n=198)*	App Group *(n=100)*	No-App Group *(n=98)*	*P* Value
Laboratory profiles, mean ± SD				
Hemoglobin (g/dL)	9.9 ± 1.9	10.1 ± 1.8	9.7 ± 2.1	.18
Hematocrit (%)	30.7 ± 6.0	31.2 ± 5.6	30.2 ± 6.3	.23
Fasting Blood Sugar (mg/dL)*	130.8 ± 62.2	118.3 ± 39.3	146.3 ± 80.1	.02
Blood Urea Nitrogen (mg/dL)	50.1 ± 19.7	50.0 ± 18.3	50.3 ± 21.1	.91
Creatinine (mg/dL)	11.2 ± 5.4	11.2 ± 4.8	11.3 ± 6.1	.85
Sodium (mEq/L)	135.5 ± 4.7	135.6 ± 4.3	135.3 ± 5.1	.61
Potassium (mEq/L)	4.0 ± 0.8	4.1 ± 0.8	4.0 ± 0.7	.39
Bicarbonate (mEq/L)	25.1 ± 3.9	25.2 ± 3.3	25.0 ± 4.4	.75
Chloride (mEq/L)	93.5 ± 13.0	93.9 ± 12.9	93.1 ± 13.2	.63
Calcium (mg/dL)	8.4 ± 1.2	8.6 ± 1.1	8.3 ± 1.2	.11
Phosphorus (mg/dL)	5.0 ± 2.0	5.0 ± 1.9	4.9 ± 2.1	.95
Albumin (g/dL)	3.1 ± 0.7	3.2 ± 0.7	3.1 ± 0.7	.53

* Tested in diabetic patients; app, application
